# Molecular diversity and migration of GABAergic neurons in the developing ventral midbrain

**DOI:** 10.1016/j.isci.2024.111239

**Published:** 2024-10-23

**Authors:** Özge Düdükcü, Divya D.A. Raj, Lieke L. van de Haar, Laurens M. Grossouw, Louisa E. Linders, Oxana Garritsen, Youri Adolfs, Nicky C.H. van Kronenburg, Mark H. Broekhoven, Troy H.W. Kapteijns, Frank J. Meye, R. Jeroen Pasterkamp

**Affiliations:** 1 Department of Translational Neuroscience, University Medical Center Utrecht Brain Center, Utrecht University, 3584 CG Utrecht, the Netherlands

**Keywords:** Molecular neuroscience, Developmental neuroscience, Cellular neuroscience

## Abstract

Dopaminergic neurons in the ventral midbrain (mDA) are surrounded by GABAergic neurons. The full extent of GABAergic neuron subtypes occupying this region and the mechanisms that underlie their development and function are largely unknown. Therefore, we performed single-cell RNA sequencing (scRNA-seq) of fluorescence-activated cell sorting (FACS)-isolated GABAergic neurons in the developing mouse ventral midbrain. Several distinct GABAergic neuron subtypes were identified based on transcriptomic profiles and spatially assigned to the ventral midbrain using *in situ* hybridization and immunohistochemistry for specific markers. A subset of GABAergic clusters that co-expressed mDA markers was studied in more detail and showed distinctive molecular, functional, and wiring properties. Finally, migration of different GABAergic neuron subtypes required netrin-1 from different cellular sources acting via distinct receptor mechanisms. Overall, our work provides insight into the heterogeneity and spatial organization of GABAergic neurons in the developing ventral midbrain and begins to dissect the mechanisms that underlie their development.

## Highlights

• scRNA-seq identifies GABAergic neuron subtypes in the developing ventral midbrain
• Developing GABA-DA neurons show specific molecular, functional, and wiring properties
• Netrin-1 controls the migration of several ventral midbrain GABAergic neuron subtypes
• Distinct netrin-1 cellular sources and receptors underlie subtype-dependent migration

## Graphical abstract

**Figure:**
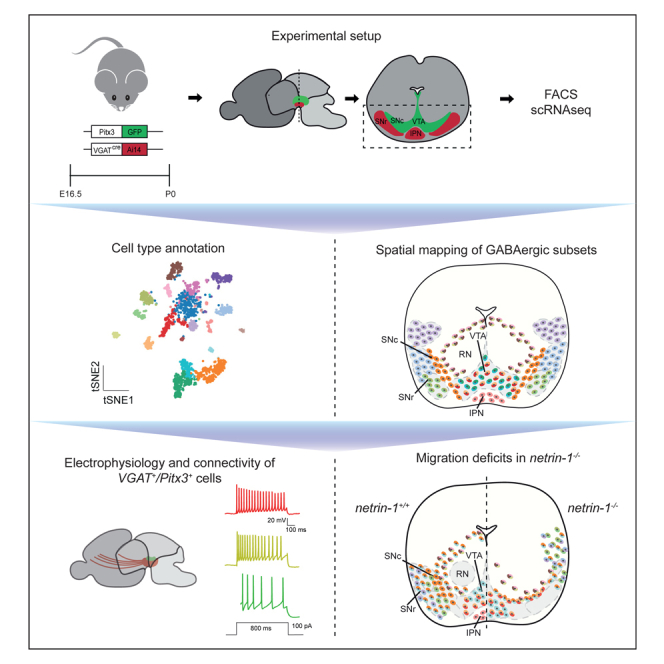

## Introduction

The midbrain dopamine (mDA) system is associated with various groups of GABAergic neurons. These GABAergic neurons not only function as local inhibitory interneurons for mDA neurons but can also project over long distances to connect to other brain regions. These regions in part overlap with known targets of mDA projections, including the prefrontal cortex, basal ganglia, and other limbic areas.^1^^,^^2^^,^^3^^,^^4^ In the ventral midbrain, GABAergic neurons can regulate mDA neuron activity in the substantia nigra pars compacta (SNc) and ventral tegmental area (VTA). For example, GABAergic projection neurons in the substantia nigra pars reticulata (SNr) inhibit mDA neurons through their axon collaterals. Through these complex connections, GABAergic neurons in the midbrain contribute to several complex behaviors. Further, changes in neurons located in the ventral midbrain have been linked to different psychiatric disorders and constitute interesting therapeutic targets.^5^^,^^6^^,^^7^^,^^8^^,^^9^^,^^10^^,^^11^^,^^12^^,^^13^ The ability of ventral midbrain neurons to underpin a disproportionally large number of physiological functions and diseases is likely explained by their heterogeneous nature. Recent single cell profiling studies reveal several molecularly distinct subtypes of GABAergic neurons in the midbrain.^12^^,^^14^^,^^15^^,^^16^^,^^17^^,^^18^^,^^19^ However, precisely which molecular GABAergic neuron subtypes flank or occupy the developing mDA system as well as their connectivity patterns remains poorly understood.

During development, GABAergic neurons destined for the ventral midbrain originate from two different progenitor domains, in the diencephalon/midbrain or hindbrain. The initial fate of these neurons is dictated by the highly regulated expression of specific transcription factors.^2^^,^^12^^,^^16^^,^^20^^,^^21^^,^^22^ Several lines of experimental evidence suggest that subsequent developmental steps also require subtype-specific mechanisms and that different neuron subtypes have distinct properties. For example, while some regions containing GABAergic neurons are penetrated by the dendrites of mDA neurons (e.g., SNr), others are not (e.g., interpeduncular nucleus [IPN]).^23^^,^^24^^,^^25^ Further, different GABAergic neuron subtypes appear to be generated at distinct developmental stages. For example, GABAergic neurons of the midbrain reticular formation (MRF) are generated at embryonic day E10.5, while SNr and VTA GABAergic neurons arise at E11.5.^26^^,^^27^ Migration of GABAergic neurons into the ventral midbrain may also require subtype-specific mechanisms as axon-derived netrin-1, a secreted axon guidance protein, is responsible for positioning GABAergic neuron into the rostral but not caudal SNr.^28^ However, the molecular mechanisms that control the positioning of other GABAergic subtypes in the ventral midbrain or that regulate additional aspects of their development remain largely unknown.

Here, we perform single-cell transcriptional profiling of VGAT^+^ GABAergic neurons in the developing mouse ventral midbrain at key developmental stages for their migration and positioning. Using these data, we identify and characterize different neuron subtypes within the developing ventral midbrain GABAergic population and characterize the long-range axonal projection patterns and electrophysiological properties of subtypes co-expressing GABAergic and mDAergic markers. Furthermore, we show that the guidance molecule netrin-1 is responsible for the migration and positioning of multiple distinct GABAergic neuron subtypes by being provided from different cellular sources and by acting through different receptors. Our findings begin to address the molecular profile and connectivity pattern of specific GABAergic neuron subtypes and the molecular cues that instruct their development. Together, these data constitute a framework for future studies on GABAergic ventral midbrain development in health and disease.

## Results

### scRNA-seq identifies GABAergic neuron subtypes in the developing ventral midbrain

To study the heterogeneity of GABAergic neurons in and around the developing mDA system during the period of GABAergic neuron migration, single-cell RNA sequencing (scRNA-seq) was performed on cells isolated from *VGAT-Cre:Ai14:Pitx3-GFP* mice. In these mice, *VGAT-Cre:Ai14* labels all GABAergic neurons, while *Pitx3-GFP* predominantly marks the mDA system to ensure the precise dissection of GABAergic neurons in and around the mDA system.

**Figure 1:**
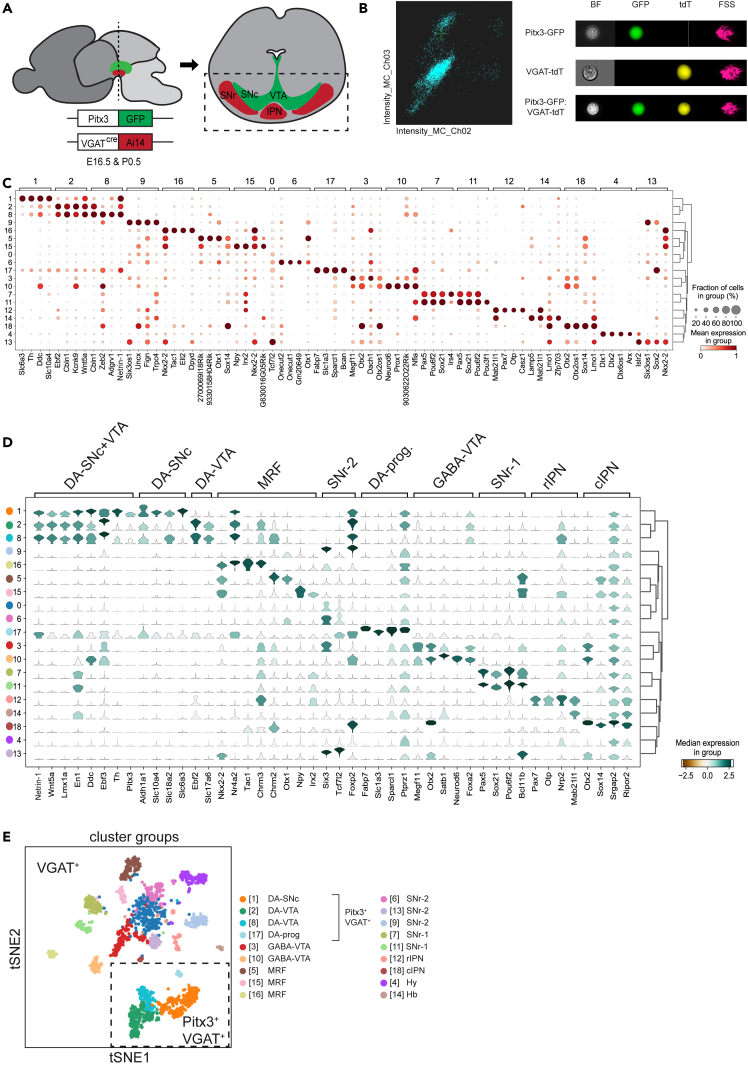
scRNA-seq identifies GABAergic neuron subtypes in the developing ventral midbrain (A) Schematic representation of the experimental design. Dashed line indicates location of the section shown at the right. E16.5 or P0.5 *VGAT-Cre:Ai14:Pitx3-GFP* mice were used for the purification of GABAergic neurons in the ventral midbrain. GFP signal allowed dissection of the mDA system. Boxed area indicates the ventral midbrain region cut out for the tissue dissociation. IPN, interpeduncular nucleus; SNc, substantia nigra pars compacta; SNr, substantia nigra pars reticulata; VTA, ventral tegmental area. (B) Imagestream analysis of tdTomato (tdT)^+^GFP^+^ labeled cells. Left: fluorescent particle detection by scatter intensity. Right: representative images of fluorescent labeled single cells (GFP, tdT, or GFP and tdT co-expressing). BF, brightfield; FSS, Forward/Side Scatter. (C) Dot-plot showing scaled and normalized expression of the top 4 differentially expressed genes (DEGs) per cluster (except for cluster 0) calculated by Wilcoxon rank-sum test after filtering (minimum normalized expression within cluster = 0.25, maximum normalized expression in other groups = 0.5, minimum fold change = 2). Disk size indicates positive fraction of cells within the group (%). (D) Stacked violin plot showing expression of selected marker genes in different clusters annotated by region in the ventral midbrain. cIPN, caudal IPN; DA-prog, DA progenitors; Hy, hypothalamus; Hb, hindbrain; MRF, midbrain reticular formation; rIPN, rostral IPN. (E) Annotated t-SNE embedding showing VGAT^+^ and VGAT^+^Pitx3^+^ (boxed area) clusters. VGAT^+^Pitx3^+^ cells represent approximately 2% of VGAT^+^ cells but are overrepresented in the study due to more extensive sampling to aid their analysis. See STAR Methods for more details. See Figure S1, Table S1.

Cells were collected at two developmental stages, E16.5 and P0.5 (Figure 1A). Migration of GABAergic neurons into the ventral midbrain starts around E14.5 leading to the occupation of nuclei, such as the SNr and the IPN at E16.5^28^ (Figure S1A). As migration of GABAergic neurons to the IPN continues until postnatal stages,^29^ samples were also collected at P0.5 (Figure S1A). Fluorescence-activated cell sorting (FACS) was performed to isolate tdTomato^+^ (tdT^+^) and tdT^+^/GFP^+^ cells. Image stream analysis confirmed the presence of cells co-expressing tdT and GFP (Figures 1B and S1B).

FACS-based CEL-seq2 scRNA-seq resulted in transcriptional profiles of 3,455 cells expressing 32,787 genes. After quality control, a total of 2,042 cells expressing 25,122 genes were used for subsequent analysis (see STAR Methods for more details).^30^ Louvain clustering revealed 19 distinct clusters, of which 4 contained tdT^+^/GFP^+^ co-expressing cells (Figures S1C–S1G).^31^ For the cluster analysis, data from both development stages were combined as no clear differences in transcriptomic profiles were observed at E16.5 and P0.5 (Figure S1D). This approach is line with other recently published developmental scRNA-seq studies.^14^^,^^32^^,^^33^^,^^34^ To facilitate more in-depth analysis of tdT^+^/GFP^+^ co-expressing neurons, these cells were collected separately and pooled in the Louvain cluster analysis. As a result, these neurons are relatively more abundant in the scRNA-seq data. Visualization of the top differentially expressed genes (DEGs) per cluster revealed that most clusters were defined by a combination of marker genes rather than unique marker genes (Figures 1C and S1H; Table S1). Based on expression of previously published marker genes,^14^^,^^29^^,^^33^^,^^35^^,^^36^^,^^37^ we annotated 15 GABAergic neuron clusters and 4 clusters co-expressing GABAergic and mDAergic markers. The annotated clusters localized to the SNc (cluster 1), SNr (6, 7, 9, 11, 13), VTA (2, 3, 8, 10), MRF (5, 15, 16), IPN (12, 18), hypothalamus (Hy; 4), or hindbrain (Hb; 14). Cluster 0 expressed GABAergic marker genes but could not be assigned to a specific region (Figures 1D, 1E, and S1C). To further study the spatial localization of some of these clusters, immunohistochemistry and *in situ* hybridization (if no specific antibodies were available) were performed to localize distinguishing markers in the ventral midbrain at different rostral-to-caudal positions. In the subsequent sections, the results for different regions are shown.

#### GABAergic neuron subtypes in the SNr

**Figure 2:**
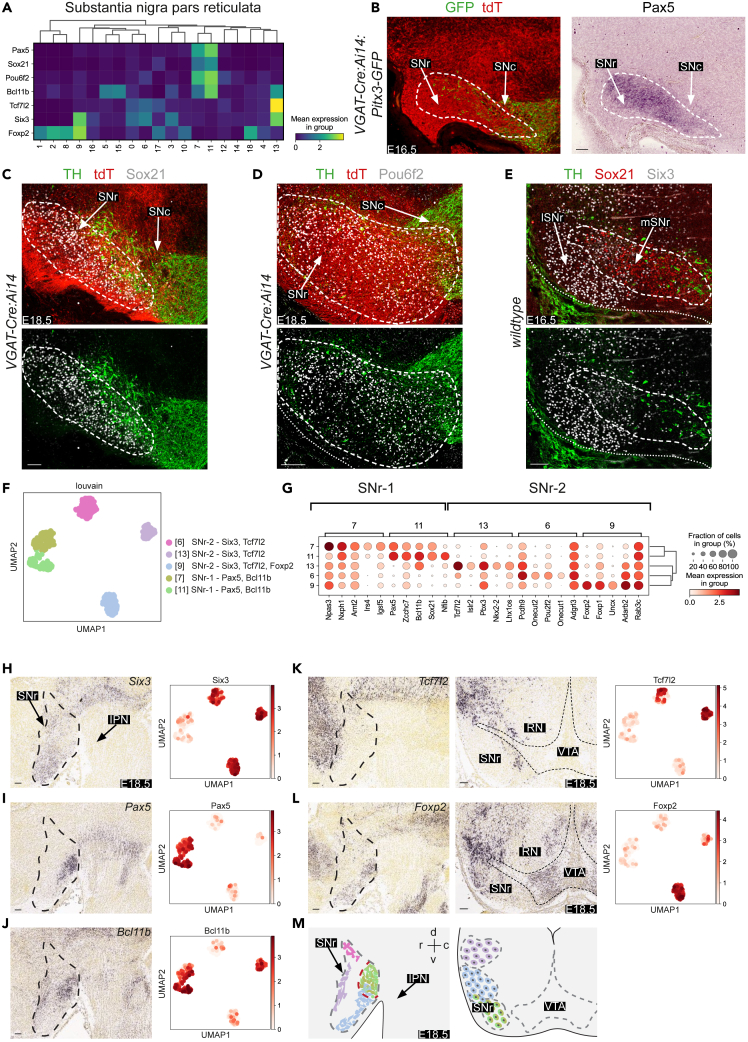
GABAergic neuron subtypes in the substantia nigra pars reticulata (A) Matrix plot showing the mean expression levels of the top ranked markers of SNr clusters (from Figure 1). (B) *In situ* hybridization for *Pax5* combined with immunohistochemistry for GFP or tdTomato (tdT) on coronal sections of the E16.5 ventral midbrain of *VGAT-Cre:Ai14:Pitx3-GFP* mice. Dashed line in B-E and H-L indicate substantia nigra pars reticulata (SNr). SNc, substantia nigra pars compacta. (C–E) Double immunohistochemistry for tdT, tyrosine hydroxylase (TH, to mark the mDA system) and the indicated SNr markers on coronal sections of the E18.5 (C and D) or E16.5 (E) ventral midbrain of *VGAT-Cre:Ai14* or wild-type mice. Each staining was performed on >3 embryos with similar results. lSNr, lateral SNr; mSNr, medial SNr. (F) UMAP embedding showing the identified SNr clusters following subclustering as detected by the Louvain algorithm per single cell and a few (sub)cluster marker genes. (G) Dot-plot showing the mean normalized expression of the top 5 ranked markers of the 5 subclustered SNr subtypes, disk size indicates positive fraction of cells within the group (%). (H–L) Spatial gene expression analysis of SNr markers. For each panel (and marker) UMAP embedding of the selected SNr clusters is shown at the right and *in situ* hybridization of sagittal sections of the E18.5 ventral midbrain at the left (from the Allen Brain Atlas; rostral is to the left). For (K) and (L), coronal sections are also shown. Dashed line indicates SNr in sagittal sections and mDA system in coronal sections. IPN, interpeduncular nucleus; RN, red nucleus; VTA, ventral tegmental area. (M) Schematic representations of the location of different SNr clusters. Left schematic, rostral is to the left. Right schematic is a coronal view. Color coding as in (F). c, caudal; d, dorsal; r, rostral; v, ventral. Scale bar, 100 μm. See Figure S2.

Our scRNA-seq data identified distinct SNr clusters, either marked by the SNr marker *Six3* (cluster 6, 9, and13) or *Pax5* (cluster 7 and 11).^14^^,^^15^^,^^16^^,^^35^^,^^38^ *Tcf712* and *Foxp2* were strongly expressed in some of the *Six3*^*+*^ clusters (6 and 13), and *Sox21* and *Pou6f2* were strongly expressed in *Pax5*^*+*^ clusters (7 and 11) (Figure 2A). *Bcl11b* was a shared marker of several of these subtypes (cluster 7, 11, and 13) (Figure 2A). Immunohistochemistry combined with *in situ* hybridization or double immunohistochemistry on embryonic *VGAT-Cre:Ai14:Pitx3-GFP* mice were used to confirm expression of some of these markers, i.e., *Pax5*, Sox21, and Pou6f2, in the tdT^+^ GABAergic area of the SNr (Figures 2B–2D). Six3 expression was reported previously^28^^,^^38^ and its analysis revealed an interesting SNr subregion-specific distribution of some marker genes. Six3 was predominantly expressed in the lateral SNr (lSNr), while Sox21 expression was localized closer to the SNc in the medial SNr (mSNr) (Figures 2C and 2E). To examine whether further subregions could be identified, the different SNr clusters (6, 7, 9, 11, and 13) were subclustered and top marker genes were identified (Figures 2F and 2G). Hierarchal clustering of the pairwise correlation between coupling *Z* scores was performed, which highlighted subgroups of clusters. With this process, noise from other clusters was eliminated, the signal of SNr clusters enhanced, and a relative comparison exclusively on SNr signals conducted. Spatial analysis of a selection of the markers using the Allen Brain Atlas (ABA) showed that at E18.5 the *Six3* clusters could be divided into *Six3*^*+*^*Tcf7l2*^*+*^ and *Six3*^*+*^*Foxp2*^*+*^ subclusters with a more dorsal or ventral location, respectively (Figures 2H, 2K, and 2L). Further, *Six3* and *Pax5* expression patterns were largely non-overlapping at E18.5 with prominent *Pax5* signals in the ventro-caudal SNr and *Six3* staining in other parts of the SNr (Figures 2H and 2I). *Bcl11b* labeled the *Pax5*^*+*^ area and a ventral *Six3*^*+*^ subcluster (Figures 2H–2J). Part of the *Pax5*^*+*^ region was also labeled by *Sox21*, *Pou6f2*, *Irs4*, and *Pou3f2* (Figures 2A, 2G, and S2A). Further, molecularly defined subregions of the SNr could still be detected at postnatal stages (Figures S2B and S2C). However, beyond P14 signals were weaker and the density of labeled cells decreased.

In summary, our scRNA-seq analysis identified two large, distinct GABAergic clusters within the developing SNr, expressing *Six3*^*+*^ or *Pax5*^*+*^, in addition to further molecular subgroups within these clusters with distinct ventro-lateral and rostro-caudal locations (Figure 2M).

#### GABAergic neuron subtypes in the IPN and MRF

**Figure 3:**
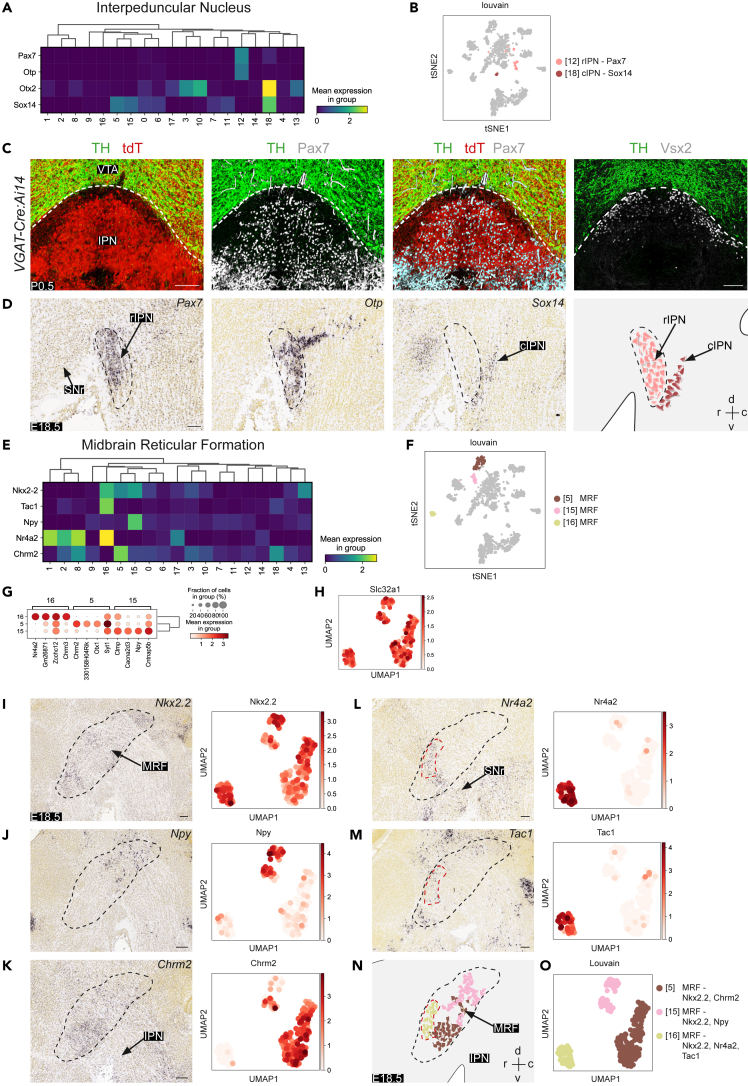
GABAergic neuron subtypes in the interpeduncular nucleus and midbrain reticular formation (A) Matrix plot showing the mean expression levels of the top ranked markers of the interpeduncular nucleus (IPN) clusters (from Figure 1). (B) t-SNE embedding showing the identified IPN clusters in the context of all identified clusters. (C) Immunohistochemistry for tyrosine hydroxylase (TH), tdTomato (tdT), and/or Pax7 or Vsx2 on coronal sections of the IPN of P0.5 *VGAT-Cre:Ai14* mice. Dashed line indicates border between ventral tegmental area (VTA) and IPN. Each staining was performed on >3 embryos with similar results. (D) *In situ* hybridization for *Pax7*, *Otp*, and *Sox14* in sagittal sections of E18.5 wildtype mice (Allen Brain Atlas). Schematic on the right summarizes the location of the different IPN clusters in a sagittal view. Dashed line indicates rostral (r) IPN.; cIPN, caudal IPN; d, dorsal; c, caudal; v, ventral. (E) Matrix plot showing the mean expression of the top ranked markers of the midbrain reticular formation (MRF) clusters (from Figure 1). (F) t-SNE embedding highlighting the identified MRF clusters in the context of all identified clusters. (G) Dot-plot showing normalized expression of the top 4 ranked markers of the 3 subclustered MRF subtypes. Disk size indicates positive fraction of cells within the group (%). (H) UMAP embedding of *Slc32a1* expression in the MRF subclusters. (I–M) Spatial gene expression analysis of MRF markers. For each panel (and marker) UMAP embedding of the selected MRF cluster(s) is shown at the right and *in situ* hybridization of sagittal sections of the E18.5 ventral midbrain at the left (from the Allen Brain Atlas; rostral is to the left). Black dashed line indicates the MRF. Red dashed line indicates location of MRF cluster 16. (N) Schematic representation of the location of different MRF clusters. Color coding as in (O). (O) UMAP embedding showing the identified MRF clusters following subclustering as detected by the Louvain algorithm per single cell and a few (sub)cluster marker genes. Scale bar, 100 μm. See Figure S3.

The IPN is located ventral of the VTA and is predominantly composed of GABAergic neurons but also contains serotonergic and glutamatergic cells.^39^^,^^40^^,^^41^^,^^42^ The dissection of the ventral midbrain in this study included part of the IPN and as a result two distinct IPN clusters were identified marked by expression of *Pax7* or *Otp* (cluster 12) and *Sox14* (cluster 18) (Figures 3A and 3B). Immunohistochemistry on sections of P0.5 *VGAT-Cre:Ai14:Pitx3-GFP* mice showed expression of Pax7 in the tdT^+^ rostral IPN (Figure 3C). The *VGAT*^*-*^ area between the VTA and the GABAergic IPN was positive for Vsx2 and has been reported to contain glutamatergic neurons (Figure 3C).^22^ *In situ* hybridization showed *Otp* and *Sox14* staining in the rostral and caudal IPN, respectively^43^ (Figure 3D). Due to the small number of cells in clusters 12 and 18, no subclustering was performed.

The MRF is located medially in the midbrain around the red nucleus (RN) and dorsal of the SNc. The MRF contained different GABAergic clusters (5, 15, and 16) that were marked by expression of *Nkx2.2* (Figures 1D and 3E; Figures S3A and S3B). While early postnatal stages (P4) showed clear *Nkx2.2* expression in the MRF, at later postnatal stages (P14-28) signals decreased and no clear region-specific patterns were found (Figures S3C–S3E). Sub-clustering of MRF clusters was performed followed by *in situ* hybridization (Figure 3G). The identified subclusters expressed *Slc32a1* (gene encoding for VGAT) confirming their GABAergic identity (Figures 3H and S3B). Cluster 5 was marked by *Chrm2* and occupied the ventrocaudal MRF (Figure 3K). Cluster 16 was marked by *Nr4a2* (also known as *Nurr1*) and *Tac1* which showed overlap in their distribution in part of the *Nkx2.2*^*+*^ area (Figures 3E, 3L, and 3M). Interestingly, different regions of the MRF showed a different neuropeptide profile. For example, cluster 16 neurons expressed *Tac1* (marking substance P neurons), while cluster 15 expressed *Npy* (Figures 3I–3M). In addition, cluster 4 was marked by *Dlx1* and identified as a hypothalamic cluster, while cluster 14 was identified as a *Lamp5*-positive hindbrain cluster (Figures S3I and S3J).

In conclusion, our analysis identified two distinct GABAergic IPN clusters located at different rostro-caudal levels (Figure 3D, bottom right panel). Further, different GABAergic neuron subtypes were found in the MRF, which all expressed *Nkx2.2* but differed in their expression of neuropeptides and other genes (e.g., *Chrm2* and *Chrm3*) (Figures 3N and 3O).

#### GABAergic neurons in the mDA system

**Figure 4:**
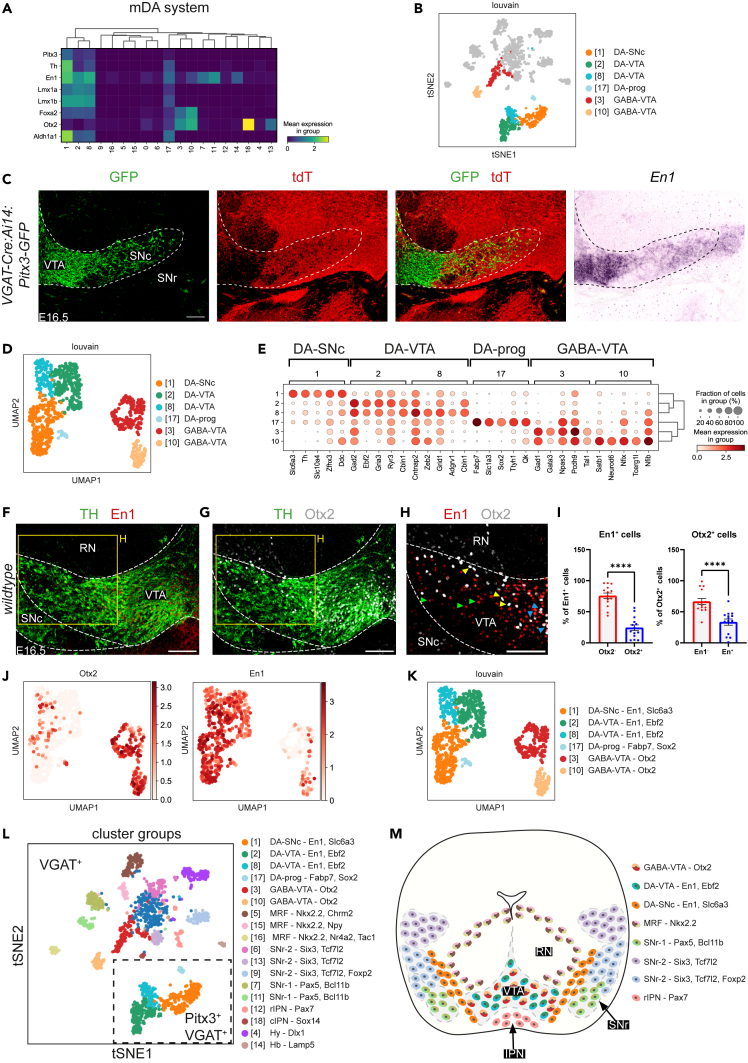
GABAergic neuron subtypes in the midbrain dopamine system (A) Matrix plot showing the mean expression of the top ranked markers of GABAergic neurons the midbrain dopamine (mDA) system (from Figure 1). (B) t-SNE embedding showing the identified clusters in the mDA system in the context of all clusters. DA-prog, DA progenitors. (C) *In situ* hybridization for *En1* combined with immunohistochemistry for GFP or tdTomato (tdT) on coronal sections of the E16.5 ventral midbrain of *VGAT-Cre:Ai14:Pitx3-GFP* mice. Dashed line indicates mDA system. SNc, substantia nigra pars compacta; SNr, substantia nigra pars reticulata; VTA, ventral tegmental area. (D) UMAP embedding showing the identified mDA system clusters following subclustering as detected by the Louvain algorithm per single cell. (E) Dot-plot showing normalized expression of the top 5 ranked markers of the 6 subclustered mDA system subtypes. Disk size indicates positive fraction of cells within the group (%). (F–H) Double immunohistochemistry for tyrosine hydroxylase (TH), Otx2, and/or En1 on coronal sections of the wild-type E16.5 mDA system. Dashed line demarcates the mDA system. Yellow boxed area is shown at higher magnification in H. RN, red nucleus. *n* = 4, 2 slices per brain. (I) Quantification of the number of En1^+^/Otx2^+^ and Otx2^+^/En1^+^ cells in the lateral portion of the VTA (H). Per mouse two sections obtained from two different rostral-caudal levels were used and both sides of the DA system were analyzed (*n* = 4 mice). Data points indicate sections and data are presented as means ± SEM. Normality tested with Shapiro-Wilk, two-tailed t test, ∗∗∗∗*p* < 0.0001. (J) UMAP embedding showing the expression levels of the selected marker genes *Otx2* and *En1*. (K) Annotated UMAP embedding of the mDA system clusters following subclustering with (sub)cluster specific markers. (L) Annotated t-SNE embedding showing VGAT^+^ and VGAT^+^Pitx3^+^ (boxed area) clusters in the ventral midbrain including (sub)cluster markers genes. This panel is identical to (Figure 1E). (M) Schematic overview of the spatial localization of the different GABAergic clusters in the late embryonic ventral midbrain. Color coding as in (L). Scale bar, 100 μm. See Figure S3.

In addition to regions surrounding the mDA neuron pool, GABAergic neuron subtypes were identified in the mDA system (SNc and VTA) (Figure 1E). These clusters expressed *Slc32a1* and a few co-expressed *Slc32a1* and *Pitx3*. These double-labeled cells (tdT^+^/GFP^+^) were isolated separately by FACS and RNA-seq was performed (Figures 1B–1D, S1C, and S1D). Using a selection of transcription factors that mark the mDA system, 6 clusters were assigned to the SNc and VTA (1–3, 8, 10, and 17). Four of these clusters co-expressed mDAergic and GABAergic markers (1 [DA-SNc], 2 [DA-VTA], 8 [DA-VTA], and 17 [DA-prog]) (Figures 1D, 1E, 4A, and 4B). This is in line with previous studies showing the presence of GABAergic/mDAergic neurons in the adult midbrain.^17^^,^^33^^,^^44^ To confirm these data, *in situ* hybridization was performed for a selected marker, *En1*, which is expressed in clusters 1, 2, 8, and 17. *En1* was expressed in the mDA system in the area of tyrosine hydroxylase (TH) and VGAT (tdT) expression (Figures 4C and S3F–S3H). To explore further heterogeneity, the 6 clusters that were originally assigned to the mDA system were sub-clustered (Figure 4D). GABAergic neurons in the mDA system could be grouped into subdomains that were classified by the combinatorial expression of specific molecular factors (Figure 4E) (DA-SNc, DA-VTA, DA-prog, and GABA-VTA). Interestingly, analysis of the top ranked marker genes of cluster 17 identified this small subset as progenitor cells of neurons in the GABAergic/mDAergic clusters (Figure 4E). Further, GABA-VTA clusters 3 and 10 were labeled by *Otx2*, while DA-VTA clusters 2 and 8 had less prominent *Otx2* expression but prominent *En1* labeling (Figures 4A and 4F–4I). Immunohistochemistry confirmed that both Otx2 and En1 were expressed in the lateral portion of the VTA (Figures 4F–4H). Generally, Otx2 cells lacked TH signal, whereas En1-positive cells expressed TH, consistent with the clustering data. Interestingly, only a small number of cells co-expressed Otx2 and En1 in this region (Figure 4I).

In conclusion, our data identify different GABAergic neuron subtypes in the developing mDA system, in part co-expressing GABAergic and mDAergic markers (Figures 4J–4M).

### Electrophysiological properties and wiring patterns of VGAT^+^Pitx3^+^ neurons

**Figure 5:**
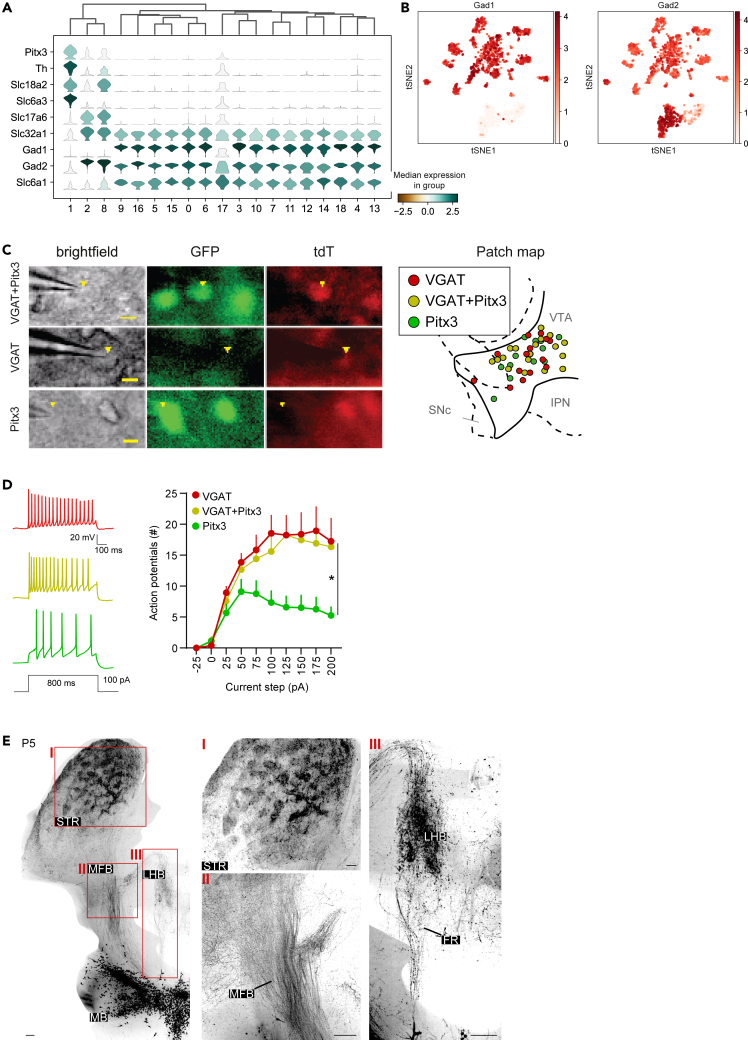
VGAT^+^Pitx3^+^ neurons display specific electrophysiological profiles and axon projections (A) Stacked violin plots showing expression of selected genes involved in the synthesis and transport of DA, glutamate and GABA in the different ventral midbrain GABAergic clusters. (B) t-SNE embedding showing expression of *Gad1* and *Gad2*. See Figure 1E for annotation. (C) Electrophysiological characteristics of VGAT^+^(tdT^+^), VGAT^+^Pitx3^+^ (tdT^+^GFP^+^), and Pitx3^+^ subsets (GFP^+^) in the midbrain. Example of patched neurons from different identities. Representative images of patched neurons (indicated by yellow triangle) in bright field, in GFP and tdTomato (tdT) channels, for conditions of VGAT^+^Pitx3^+^ neurons (top), single VGAT^+^ neurons (middle) and single Pitx3^+^ neurons (bottom). Scale bar, 10 μm. Right: Map of patched cells across the conditions in the ventral tegmental area (VTA). IPN, interpeduncular nucleus; SNc, substantia nigra pars compacta. (D) VGAT^+^Pitx3^+^ neurons and single VGAT^+^ neurons show similar firing patterns, while Pitx3^+^ neurons show reduced firing (n VGAT^+^ = 13 n Pitx3^+^ = 12, n double VGAT^+^Pitx3^+^ = 20, RM ANOVA, F(2,41) = 4.2, *p* = 0.022). Error bars represent ±SEM. (E) P5 *VGAT-Cre:Pitx3-FlpE:Ai65* mice were immunostained for tdT, optically cleared and imaged in a horizontal plane using fluorescent light sheet microscopy (FLSM). Left: representative max projection of 500 μm sections of a hemisphere from a horizontal plane z stack. Boxed areas are shown at higher magnification at the right. Images at the right show axons in striatal (STR) patches (I), the medial forebrain bundle (MFB) (II) and the lateral habenula (LHb) (III). HY, hypothalamus, MB, midbrain; FR, fasciculus retroflexus. Stainings were performed on >3 mice with similar results. Scale bar, 100 μm. See Figures S4 and S5; Tables S2 and S3.

Our scRNA-seq analysis of the ventral midbrain of *VGAT-Cre:Ai14:Pitx3-GFP* mice identified several GABAergic neuron subtypes in and around the mDA neuron pool (Figures 4L and 4M). Several of these subtypes co-expressed markers of mDAergic and GABAergic neurons, which is in line with the observation that in the adult mDA system subsets of neurons can synthesize and/or release more than one neurotransmitter.^45^^,^^46^^,^^47^ To examine whether such combinatorial expression of neurotransmitters could already be detected at developmental stages, expression of genes involved in the synthesis and transport of different neurotransmitters was studied in the clusters identified in this study (Figure 1E). This analysis confirmed that several clusters expressed markers corresponding to different neurotransmitter systems (Figures 5A and 5B). For example, clusters 1, 2, 8, and 17 not only expressed GABAergic markers but also genes implicated in DA synthesis and transport (*TH*, *Slc6a3* [encoding DAT], *Ddc* and *Slc18a2* [encoding VMAT2]). *TH* and *Ddc*, both involved in DA synthesis, and *Gad1* and *Gad2*, involved in GABA synthesis, showed distinct expression in different sets of clusters. *Gad1* was expressed in all VGAT^+^ clusters (in the SNr, IPN, MRF and to a lesser extent in the SNc), whereas *Gad2* was more strongly expressed in VGAT^+^/Pitx3^+^ clusters (most prominently in VTA clusters 2 and 8), as well as in all VGAT^+^ clusters. *In situ* hybridization confirmed this differential pattern of expression at adult stages (Figures S4A and S4B).

To establish whether the molecularly distinct GABAergic and GABAergic/mDAergic neuron subtypes identified in our study also display different functional properties, whole-cell current clamp recordings were performed on VGAT^+^, Pitx3^+^, and VGAT^+^Pitx3^+^ co-expressing neurons in the VTA of postnatal *VGAT-Cre:Ai14:Pitx3-GFP* mice (between P3 and P12). VGAT^+^Pitx3^+^ neurons were defined as tdT^+^/GFP^+^, VGAT^+^ neurons as tdT^+^/GFP^−^, and Pitx3^+^ neurons as tdT^−^/GFP^+^. The three populations were equally distributed in terms of age of the recorded animals (average P + SEM; VGAT^+^ = 6.9 + 0.69, Pitx3^+^ = 6.8 + 0.96, VGAT^+^Pitx3^+^ = 7.0 + 0.48, Table S2). Moreover, the postnatal age at the time of patching did not significantly correlate with the neurophysiological properties that were assessed in the three cellular populations (Table S3). Recordings of the three subtypes were performed throughout the VTA (Figure 5C). These measurements revealed that VGAT^+^Pitx3^+^ and VGAT^+^ neurons fired more action potentials in response to current injections as compared to Pitx3^+^ neurons (Figure 5D). Further, VGAT^+^Pitx3^+^ and VGAT^+^ neurons had a lower action potential threshold as compared to Pitx3^+^ neurons (Figure S4B). Resting membrane potential, membrane resistance, capacitance, and voltage sag were not different between the three populations (Figures S4C–S4F). Thus, neurons in the VTA that express GABAergic or both mDAergic and GABAergic markers exhibit different electrophysiological firing properties as compared to mDAergic neurons in their immediate environment.

As VGAT^+^Pitx3^+^ neurons displayed distinct electrophysiological properties as compared to Pitx3^+^ neurons, we next assessed their axonal wiring pattern using P5 *Pitx3-FlpE:VGAT-Cre:Ai65* mice. In *Pitx3-FlpE* mice, a FlpE expression cassette replaces exons 2, 3, and in part exon 4 of the *Pitx3* gene, allowing for specific labeling of Pitx3-expressing neurons (Figure S5A). In line with the scRNA-seq data (Figure 1E), a subset of TH^+^ mDA neurons expressed tdT (VGAT + Pitx3) in the SNc and VTA (Figures S5B–S5D). Vice versa, a subset of neurons in the ventral midbrain, particularly in the rostral linear nucleus, expressed VGAT and Pitx3 but not TH (tdT^+^/TH^−^ cells) (Figures S5A and S5B). iDISCO^48^ was used to optically clear whole brains following whole-mount immunostaining followed by fluorescent light sheet microscopy (FLSM). tdT^+^ cell bodies were confined to the ventral midbrain (SNc, VTA, rostral linear nucleus), confirming the specificity of the intersectional strategy, and prominent axon projections to the forebrain were detected (Figure 5E). The overall wiring patterns of tdT^+^ axons were similar to those described for mDA neurons as detected by for example TH immunostaining or in *Pitx3-GFP* mice, with innervation of targets such as striatum or the habenula. However, both the medial forebrain bundle (MFB) and striatum were less broadly populated by tdT^+^ axons as compared to TH^+^ axons (Figures S5E–S5G). Interestingly, striatal patches appeared enriched for both TH^+^ axons and tdT^+^ axons, while tdT^+^ innervation of the habenula was restricted to the lateral habenula (LHB) as compared to broader TH labeling (Figures 5E and S5E). We observed a few tdT^+^TH^−^ axons in additional regions, including the cortex, septum, and thalamus, in line with the known projection patterns of GABAergic neurons. No regions beyond those shown in the figures exhibited such axonal labeling. These data indicate that the majority of VGAT^+^Pitx3^+^ neurons project to known mDA target areas, while a subset of VGAT^+^Pitx3^+^TH^−^ neurons projecting to non-mDA target areas.

Together, these findings identify a group of molecularly distinct GABAergic neurons in the developing mDA system which express mDAergic markers (e.g., *Th* and *Pitx3*) and have distinct functional properties (i.e., compared to mDAergic neurons) and distinctive axonal innervation patterns.

### Netrin-1 instructs the positioning of different ventral midbrain GABAergic subtypes

**Figure 6:**
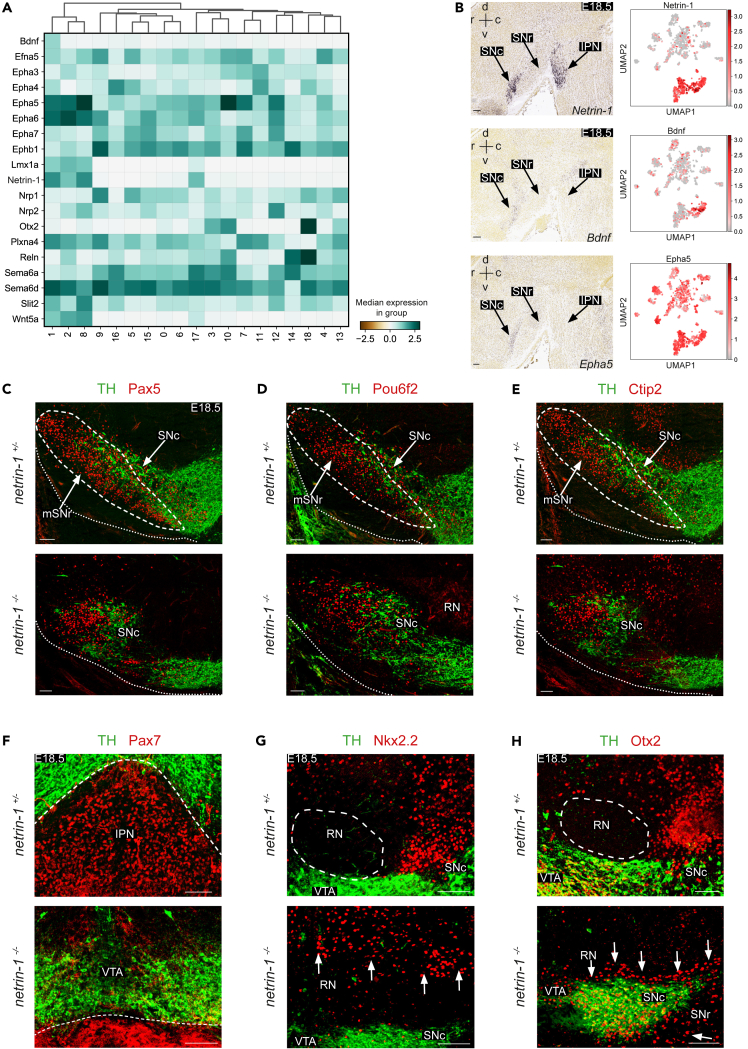
Netrin-1 instructs the positioning of different ventral midbrain GABAergic neuron subtypes (A) Heatmap showing the expression of axon guidance cues (GO:0008046, GO:0007411) in the ventral midbrain GABAergic neuron clusters. (B) Expression of selected axon guidance cues *netrin-1*, *Bdnf* and *Epha5*. Left: *In situ* hybridization for the selected axon guidance cues in sagittal sections of E18.5 ventral midbrain (from the Allen Brain Atlas; rostral is to the left). Right: UMAP embedding showing the expression of the selected axon guidance cues in the ventral midbrain GABAergic clusters. Please see Figure 1E for cluster annotations. SNc, substantia nigra pars compacta; SNr, substantia nigra pars reticulata; IPN, interpeduncular nucleus. (C–H) Double immunohistochemistry for tyrosine hydroxylase (TH) and the indicated proteins on coronal sections of the ventral midbrain of E18.5 *netrin-1*^*+/−*^ (control) and *netrin-1*^*−/−*^ mice. Each staining was performed on >3 embryos with similar results. RN, red nucleus; VTA, ventral tegmental area. Dashed line indicates SNr (C–E), border VTA and IPN (F), or RN (G and H). Arrows indicate aberrantly localized cells. mSNr, medial SNr. Scale bar, 100 μm. See Figure S6.

The molecular mechanisms that direct the migration of ventral midbrain GABAergic neurons during development are poorly understood. Our scRNA-seq data of GABAergic neurons isolated at developmental stages during which their migration occurs provide an opportunity to identify molecular factors that may regulate these migratory events. As axon guidance proteins are well-known for their role in neuron migration,^49^ the expression of these cues was examined in the different GABAergic clusters. This revealed broad expression for some cues (e.g., *Ephb1* and *Sema6d*) and more subtype-dependent expression for others (e.g., *netrin-1*, *Bdnf*, and *Epha5*) (Figures 6A and 6B). Interestingly, *netrin-1*, *Bdnf*, *Lmx1a*, and *Wnt5a* were almost exclusively expressed in the VGAT^+^Pitx3^+^ clusters. As our previous work had shown that netrin-1 is required for the migration of Six3^+^ GABAergic neurons to the SNr,^28^ we explored potential roles for this cue in the positioning of other GABAergic subtypes in the ventral midbrain.

First, the spatial localization of the GABAergic markers *Gad1*, *Gad2*, and *Tal1* was assessed in E18.5 *netrin-1*^*−/−*^ mice and littermate controls (*netrin-1*^*+/−*^ and *netrin-1*^*+/+*^ embryos were used with similar results) to establish the overall effect of netrin-1 depletion on the distribution of GABAergic neurons (Figures S6A–S6C). In *netrin-1*^*+/−*^ mice, *Gad1*^*+*^ and *Gad2*^*+*^ neurons were located around the RN and in the SNc, whereas *Gad2*^*+*^ was also found in the VTA. In *netrin-1*^*−/−*^ embryos, the mDA neuron pool had flattened, as reported previously.^28^^,^^50^ *Gad1*^*+*^ neurons mislocalized in the RN and dorsal of the mDA neuron pool in *netrin-1*^*−/−*^ embryos (Figure S6A). *Gad2* expression around the RN was intact but some *Gad2*^*+*^ neurons aberrantly localized dorsal of the mDA neuron pool. Further, as the mDA system had flattened in the absence of netrin-1, the density of *Gad2*^*+*^ neurons in this structure increased (Figure S6B). *Tal1* was strongly expressed in neurons in the MRF and SN (SNc and SNr) in *netrin-1*^*+/−*^ embryos. In *netrin-1*^*−/−*^ mice, *Tal1*^*+*^ neurons invaded the RN and accumulated in specific regions of the SN (Figure S6C). Together, these data suggest that the migration of several GABAergic subtypes is affected in the ventral midbrain in the absence of netrin-1.

To study the effect of netrin-1 ablation on specific GABAergic neuron subtypes in the ventral midbrain, we first focused on the SNr. Six3^+^ GABAergic neurons rely on axon-derived netrin-1 for entering the rostral SNr during embryonic development.^28^ To test whether the migration or positioning of other SNr subtypes is also netrin-1-dependent, we assessed Pax5^+^(Six3^-^) neurons. In *netrin-1*^*+/−*^ mice, Pax5^+^ neurons were localized lateral of the TH^+^ SNc mDA neurons in the SNr (Figure 6C). However, in E18.5 *netrin-1*^*−/−*^ mice, TH^+^ neurons occupied the SNr territory and Pax5^+^ GABAergic neurons, similar to Six3^+^ neurons,^28^ accumulated more dorsally (Figure 6C). Other markers for GABAergic neurons in the SNr, such as *Pou6f2* and *Bcl11b* (Ctip2) (Figures 2D and 2J), also showed similar mislocalization in *netrin-1*^*−/−*^ mice (Figures 6D and 6E).

Next, GABAergic subtypes in the IPN and MRF were examined. Netrin-1 has been implicated in the migration of neurons to IPN,^29^^,^^51^^,^^52^ and particularly of Pax7^+^ neurons.^43^ However, the effect of netrin-1 depletion had not been studied. Analysis of *netrin-1*^*−/−*^ mice showed displacement of TH^+^ neurons and reduced *Pax7* expression confirming the role of *netrin-1* in GABAergic IPN development (Figure 6F). In control *netrin-1*^*+/−*^ mice, GABAergic neurons in the MRF expressed Nkx2.2 and were located around the RN and dorsal of the SNc (Figure 6G). In contrast, in E18.5 *netrin-1*^*−/−*^ mice, a subset of Nkx2.2^+^ neurons had invaded the RN region and Nkx2.2^+^ neurons were absent from the region immediately dorsal of the SNc (Figure 6G). In the ventral midbrain, *Otx2* is expressed in the VTA, MRF, and cIPN. In *netrin1*^*−/−*^ mice, Otx2^+^ neurons were still present in the VTA, but their organization was disrupted. Compared to control *netrin-1*^*+/−*^ mice, in which no Otx2 expression was detected outside of the VTA or in the MRF, Otx2^+^ neurons were positioned dorsal as well as ventral of the SNc and VTA in *netrin-1*^*−/−*^ mice (Figure 6H).

Together, these results indicate that netrin-1 is required for the migration of several GABAergic neuron subtypes. For example, for positioning SNr GABAergic neurons ventrolateral to the SNc, GABAergic IPN neurons ventral to the VTA, and GABAergic MRF neurons dorsal to the SNc.

### Ventral midbrain GABAergic neuron migration requires specific netrin-1 sources and receptors

**Figure 7:**
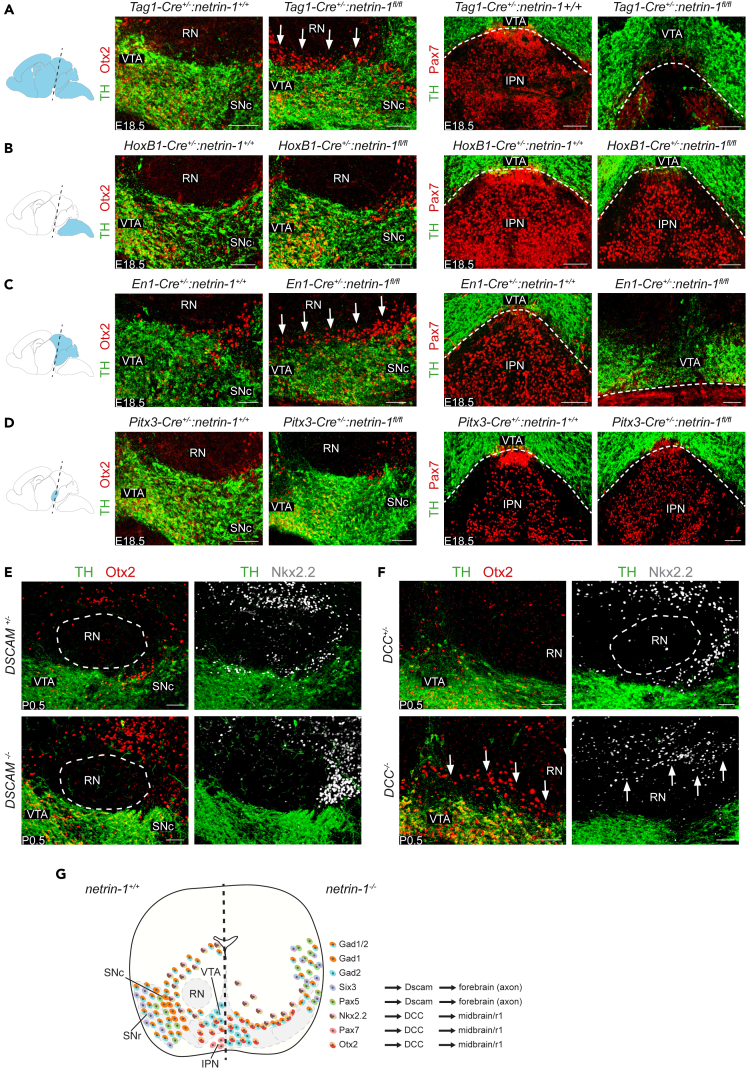
Ventral midbrain GABAergic neuron migration requires specific netrin-1 sources and receptors (A–D) Double immunohistochemistry for tyrosine hydroxylase (TH) and the indicated proteins in coronal sections of the ventral midbrain of E18.5 *netrin-1*^*f*^^*l*^^*/f*^^*l*^ mice crossed with the indicated Cre lines. Schematic at the left shows Cre expression patterns (in blue) in the different mouse Cre lines (at the right). Each staining was performed on >3 embryos with similar results. IPN, interpeduncular nucleus; RN, red nucleus; SNc, substantia nigra pars compacta; SNr, substantia nigra pars reticulata; VTA, ventral tegmental area. Dashed line indicates border VTA and IPN (A–D). Arrows indicate aberrantly localized cells. Scale bar, 100 μm. (E and F) Double immunohistochemistry for TH and the indicated proteins on coronal sections of the ventral midbrain of P0.5 down syndrome cell adhesion molecule (*DSCAM*)^*−/−*^ or deleted in colorectal cancer (*DCC*)^*−/−*^ mice and littermate controls. Ablation of netrin-1 receptors DSCAM (E) and DCC (F). Double immunohistochemistry for subsets Otx2 and Nkx2.2 for each receptor knockout. Dashed line indicates the RN. Arrows indicate aberrantly localized cells. Scale bars, 100 μm. (G) Schematic overview of changes in the localization of ventral midbrain GABAergic subtypes in *netrin-1*^*−/−*^ mice, associated netrin-1 receptors and cellular sources. Six3 data are derived from.^28^ Scale bar, 100 μm. See Figure S6.

As striatal axon-derived netrin-1 instructs Six3^+^ neurons to localize ventrolateral of the SNc,^28^ we next asked which cellular sources of netrin-1 are required for guiding other GABAergic subtypes in the ventral midbrain. Our data showed strong *netrin-1* expression in Pitx3^+^VGAT^+^ clusters (Figures 6A and 6B). *Netrin-1* expression has also been reported in the ventricular zone (VZ), and netrin-1 protein was detected in radial glia, mDA neurons, and the cerebral peduncle.^28^^,^^53^^,^^54^^,^^55^^,^^56^^,^^57^ Therefore, conditional knockout (cKO) experiments were performed to establish which netrin-1 sources are required for the migration of some of the GABAergic subtypes identified in this study. Analysis of E18.5 *Tag1-Cre:netrin-1*^*f*^^*l*^^*/f*^^*l*^ mice revealed a strong disorganization of several different GABAergic subtypes, i.e., Otx2^+^ VTA, Pax7^+^ IPN, and Nkx2.2^+^ MRF as compared to several control genotypes (Figures 7A and S6D). However, *Tag1* is expressed throughout the CNS and Tag1^+^ axons in the ventral midbrain originate from different neuronal populations throughout the brain. Therefore, in subsequent experiments Cre lines that target the hindbrain and the spinal cord (*HoxB1-Cre*), midbrain and rhombomere 1 (r1) (*En1-Cre*), or specifically mDA neurons (*Pitx3-Cre*) were used. P0.5 *HoxB1-Cre:netrin-1*^*f*^^*l*^^*\fl*^ and *HoxB1-Cre:netrin-1*^*+\+*^ mice showed a similar pattern of Otx2 and Pax7 expression in the ventral midbrain, arguing against a role for hindbrain/spinal cord-derived netrin-1 (Figure 7B). Analysis of E18.5 *En1-Cre:netrin-1*^*f*^^*l*^^*/f*^^*l*^ mice showed ectopic localization of Otx2^+^ neurons dorsal to the SNc as observed in *netrin-1*^*−/−*^ mice. Moreover, these embryos showed failure of GABAergic neuron migration into the IPN. This region was instead occupied by mDA neurons (Figure 7C). In contrast, expression of Nkx2.2 around the RN was unchanged in E18.5 *En1-Cre:netrin-1*^*f*^^*l*^^*/f*^^*l*^ mice (Figure S6E). These data show that local neurons or other cells in the midbrain or r1 are the cellular source of netrin-1 for instructing the migration of GABAergic subtypes in the VTA and IPN but not MRF. To examine whether mDA neurons comprise this local source, *Pitx3-Cre:netrin-1*^*f*^^*l*^^*/f*^^*l*^ mice were analyzed at E18.5. In these mice, no changes in the organization of Otx2^+^ neurons or the Pax7^+^ IPN were found (Figure 7D). Similarly, depletion of the mDA system in E18.5 *Pitx3-Cre*^*+/−*^*:DTA*^*+/+*^ mice did not affect the distribution of Pax7^+^ neurons in the IPN (Figure S6F). To assess whether netrin-1 derived from the habenula, a netrin-1^+^ structure that provides major afferent inputs to the IPN,^58^^,^^59^ can also contribute to the migration of Pax7^+^ GABAergic neurons into the IPN *Dbx1-Cre*^*+/−*^*:netrin-1*^*f*^^*l*^^*/f*^^*l*^ mice were analyzed. However, no differences in Pax7 distribution in the IPN of *Dbx1-Cre*^*+/−*^*:netrin-1*^*+/+*^ and *Dbx1-Cre*^*+/−*^*:netrin-1*^*f*^^*l*^^*/f*^^*l*^ mice at E18.5 were observed (Figure S6G). Thus, our data indicate that cells in the midbrain or r1 express netrin-1 to control Pax7^+^ GABAergic IPN and Otx2^+^ GABAergic neuron migration. Analysis of the SNr marker Sox21 in *FoxG1-Cre*^*+/−*^*:netrin-1*^*f*^^*l*^^*/f*^^*l*^ mice at E18.5 revealed mispositioning of Sox21^+^ neurons outside the SNr suggesting that, similar to Six3^+^ GABAergic neurons,^28^ migration of this GABAergic subtype relies on forebrain-derived netrin-1 (Figure S6H).

The effects of netrin-1 can be mediated by different cell surface receptors. For example, down syndrome cell adhesion molecule (DSCAM) is required for Six3^+^ GABAergic neurons to respond to striatal axon-derived netrin-1 in the SNr.^28^ To investigate which netrin-1 receptors are involved in the migration of different GABAergic subtypes in the ventral midbrain, we first assessed their expression. Our scRNA-seq data revealed prominent expression of deleted in colorectal cancer (*DCC*) and *DSCAM*, and weaker expression of *Unc5c*, *Unc5D*, and *Neogenin* (Figure S6I). Analysis of *DSCAM*^*−/−*^ mice at P0.5 did not reveal changes in the distribution of Pax7^+^ IPN, Nkx2.2^+^ MRF, and Otx2^+^ VTA neurons (Figures 7E and S6K). This suggests that only GABAergic neurons of the SNr rely on DSCAM.^28^ Genetic ablation of DCC has been shown to cause defects in the Pax7^+^ IPN,^43^ similar to our observations in *netrin-1*^*−/−*^ mice (Figures 6F and 7F). In addition, we found invasion of the RN by Nkx2.2^+^ neurons and ectopic Otx2^+^ neurons dorsal of the mDA neuron pool in *DCC*^*−/−*^ mice, as detected in *netrin-1*^*−/−*^ mice (Figures 6G, 6H, and 7F). However, no changes in the distribution of Sox21^+^ and Pax7^+^ neurons were found (Figures S6J and S6K). Together, these data show that distinct GABAergic subtypes in the ventral midbrain require different netrin-1 receptors for their migration and positioning, with SNr neurons requiring DSCAM,^28^ and other subtypes, such as IPN, MRF, and VTA, DCC (Figure 7G).

## Discussion

Different subtypes of GABAergic neurons reside in and around the mDA neuron pool, but how these subtypes develop, connect, and function remains incompletely understood. To address these unresolved questions, we performed single-cell transcriptional profiling of VGAT^+^ GABAergic neurons in the developing mouse ventral midbrain at key developmental stages. Our work identifies distinct GABAergic neuron subtypes in different parts of the ventral midbrain and further characterizes developing neurons that co-express GABAergic and mDAergic markers. These data were subsequently exploited to show that the axon guidance protein netrin-1 directs the migration and positioning of different GABAergic neuron subtypes, being provided by distinct cellular sources and by acting through distinct receptors (Figures 7G; Table S4). Our findings begin to unveil the molecular profile and developmental wiring pattern of specific GABAergic neuron subtypes as well as the molecular cues required for their development. Together, these data constitute a valuable resource for future studies on GABAergic ventral midbrain development in health and disease.

### GABAergic neuron diversity in the developing ventral midbrain

Recent single-cell profiling studies revealed molecularly distinct subtypes of GABAergic neurons in the midbrain.^12^^,^^14^^,^^15^^,^^16^^,^^17^^,^^18^^,^^19^ However, precisely which molecular GABAergic neuron subtypes flank or occupy the developing mDA system as well as their developmental mechanisms, functional roles, and connectivity patterns remain poorly understood. By performing FACS-based scRNA-seq on the ventral midbrain of *VGAT-Cre:Ai14:Pitx3-GFP* mice at two key developmental stages for neuron migration and positioning, we identified two large populations of GABAergic neurons. One marked by VGAT (tdT^+^) expression and one by a combination of VGAT and Pitx3 (tdT^+^/GFP^+^).

Although the developmental stages selected here did not allow us to firmly establish the developmental origin of these different GABAergic neuron subtypes, it has been proposed that ventral midbrain GABAergic neurons originate from two different progenitor domains, in the diencephalon/midbrain (rostral GABAergic neurons) or hindbrain (caudal GABAergic neurons).^12^^,^^16^^,^^20^^,^^21^^,^^22^^,^^60^ Neurons originating from the hindbrain (rhombomere 1) are dependent on the transcription factor *Tal1*.^16^^,^^22^ Our data identify *Tal1* in most GABAergic clusters, except for the VGAT^+^Pitx3^+^ (clusters 1, 2, 8, and 17) and IPN (cluster 12) clusters. This suggests different origins for distinct GABAergic neuron subtypes, i.e., hindbrain for VGAT^+^Pitx3^-^ and midbrain for VGAT^+^Pitx3^+^ neurons. Further work is needed to firmly establish these different origins. However, co-expression of VGAT with Pitx3, and other factors required for early mDA neuron development (such as En1 and Lmx1a),^61^^,^^62^ hints at a midbrain origin for VGAT^+^Pitx3^+^ neurons and suggests that in some cases mDAergic and GABAergic neurons may originate from the same neurogenic region.

VGAT^+^Pitx3^-^ neuron subtypes populated different ventral midbrain regions, such as SN, MRF, RN, and IPN, in which further heterogeneity with respect to molecular GABAergic neuron subtypes was detected. Such heterogeneity was especially evident in the SNr, a complex region ventral to the SNc harboring not only mDA neuron dendrites and GABAergic neurons but also various afferent connections and glial cells.^4^ Our data revealed two large clusters of SNr GABAergic neurons, i.e., *Six3*^*+*^*Pax5*^*-*^ or *Six3*^*-*^*Pax5*^*+*^.^15^^,^^16^^,^^28^^,^^35^^,^^38^ Clusters in the caudal SNr (clusters 7 and 11) expressed *Pax5*, but not *Six3*, while rostral ventro-lateral SNr clusters (clusters 6, 9, and 13) expressed *Six3* and not *Pax5*. Interestingly, further molecular subdivisions exist as *Six3* expression was predominantly observed in the lateral SNr, whereas *Sox21* expression was found more medially. This molecular diversity in GABAergic neurons along the rostral-caudal and medial-lateral axes of the developing SNr is in line with previous work demonstrating subregional heterogeneity in the SNr^12^^,^^22^^,^^35^ and with reported differences in the origin of rostral and caudal SNr neurons. For example, *Pax5*^*+*^*Zfpm2*^*+*^ neurons occupy caudal SNr and derive from rostral rhombomere 1, while *Six3*^*+*^ GABAergic neurons in the rostral SNr have been proposed to originate from progenitor zones in caudal midbrain.^16^^,^^20^ It is tempting to speculate that the molecular signatures of these SNr subclusters, composed by (combinatorial) expression of transcription factors, serve a role in the development of subregion-specific features. For example, there is topographically organized connectivity between the SNr and striatum. Molecular codes along the rostral-caudal axis of the SNr could help to establish this wiring pattern.^63^^,^^64^ Similarly, in rostral but not caudal parts of the SNr mDA neuron dendrites form bundles, i.e., dendrons, between GABAergic neurons.^65^ The molecular mechanism underlying these SNr subregion-specific wiring patterns remain unknown, but our molecular interrogation of SNr (sub)clusters may aid their identification. For example, rostral and caudal SNr clusters show differential expression (levels) of guidance cues and receptors, such as Ephs and ephrins.

In all, our work unveils a high level of molecular diversity both between and within developing GABAergic neuron subtypes in specific regions of the midbrain. These molecular codes may be instrumental for ventral midbrain development and, as many SNr subregion markers are detected also at postnatal and adult stages, may serve functions in the more mature midbrain.

### Developing GABAergic neuron subtypes that express mDA markers

Increasing experimental evidence shows co-existence of different neurotransmitters in mDA neurons. For example, SNc mDA neurons can release GABA through unconventional mechanisms using GAT1 for reuptake as well as VMAT2 for packaging.^46^ Further, co-expression of TH with various other neurotransmitters or proteins required for their function has been reported at the RNA and protein levels.^66^^,^^67^^,^^68^^,^^69^ Previous single cell profiling studies focusing on mDA neurons have identified clusters co-expressing *Pitx3* and *VGAT*, but these neurons have remained largely uncharacterized, especially also at developmental stages.^17^^,^^33^ Here, we further defined some of the molecular, functional, and wiring properties of these VGAT^+^Pitx3^+^ neurons. These neurons expressed several genes involved in DA synthesis (*Th*, *Slc6a3*, *Ddc*, and *Slc18a2*) as well as *Gad2*. Interestingly, comparison of the different VGAT^+^Pitx3^+^ subtypes also revealed subtype-specific differences. For example, *Aldh1a1*, involved in DA synthesis, was restricted to cluster 1, while cluster 8 showed *Slc18a2* expression and low levels of *Th* but no *Slc6a3* and only low levels of *Slc6a1*. At the functional level, we found that the electrophysiological properties of VGAT^+^Pitx3^+^ neurons in the VTA resembled those of adjacent VGAT^+^ but not Pitx3^+^ neurons. Further work is needed to integrate these molecular and cellular observations and link them to, for example, afferent and efferent connectivity patterns.

To begin to define the efferent connections of VGAT^+^Pitx3^+^ neurons, we established and applied an intersectional genetic labeling strategy in mice. This approach exploits the overlapping expression of genes to target cell populations with high selectivity.^28^ The *Pitx3-FlpE:VGAT-Cre:Ai65* mouse model facilitated specific labeling of VGAT^+^Pitx3^+^ neurons in the midbrain and their axonal connections. GABAergic neurons in the VTA can have short- and long-range axon projections^70^^,^^71^^,^^72^^,^^73^ and partially overlapping but also clearly distinct projection targets as compared to VTA mDA neurons.^69^,^71^^,^^74^ Here, we focused on long-range projections and a developmental stage (P5) at which axon pathfinding of mDA neurons has been largely concluded.^75^ FLSM of cleared *Pitx3-FlpE:VGAT-Cre:Ai65* mouse brains showed that the overall projection patterns of VGAT^+^Pitx3^+^ and TH^+^ mDA neurons are similar but also revealed several areas of more selective innervation, such as potentially enriched innervation of striatal patches and more restricted, medial, targeting of the habenula. It should be noted that tdT labeling appeared sometimes (but not always, e.g., MFB or habenula) more widespread than TH labeling in *Pitx3-FlpE:VGAT-Cre:Ai65* mice which in part may be attributed to the presence of VGAT^+^/Pitx3^+^/TH^−^ neurons in the rostral linear nucleus or the relatively strong expression of the tdT transgene as compared to the endogenous TH protein. In addition, while we here focus on P5, further work using region-specific markers and additional (developmental) stages is needed to establish the spatiotemporal dynamics of these innervation patterns.

Together, these data show that VGAT^+^Pitx3^+^ neuron subtypes are molecularly heterogeneous at early stages and share electrophysiological properties with GABAergic neurons. Further, connectivity patterns of VGAT^+^Pitx3^+^ neurons resemble those of mDA neurons but show region-specific selective innervation.

### Subtype-specific migration is regulated by specific netrin-1 sources and receptors

The development of ventral midbrain GABAergic neuron subtypes requires subtype-specific mechanisms as, for example, different subtypes have different origins, migratory routes, and synaptic partners. Previous work shows that axon-derived netrin-1 is responsible for positioning Six3^+^ GABAergic neurons into the rostral but not caudal SNr.^28^ However, the molecular mechanisms controlling the positioning of most other GABAergic subtypes in the ventral midbrain remain largely elusive. Our current study reveals a broad and tightly controlled role for netrin-1 in the migration and positioning of multiple distinct GABAergic subtypes (*Pax5*^*+*^, *Pax7*^*+*^, *Otx2*^*+*^, and *Nkx2*,*2*^*+*^) in the ventral midbrain. Although these different subtypes are present in the ventral midbrain of *netrin-1*^*−/−*^ mice their positioning is disrupted. Intriguingly, these effects are mediated different cellular sources of netrin-1 (striatal axon-derived^28^ and midbrain/r1) and require different receptors (DSCAM and DCC).^28^^,^^43^ It is currently unknown how specific neuron subtypes are able to respond to one but not another closeby source of netrin-1. Additional netrin-1 receptors are detected in GABAergic neurons (Figure S6I) and may contribute to this differential sensitivity. In addition, further work is needed to dissect more precisely the cellular source of netrin-1 for *Pax7*^*+*^, *Otx2*^*+*^, and *Nkx2.2*^*+*^ subtypes, as these sources may be spatially segregated. It is also possible that some of the defects observed are an indirect consequence of netrin-1 deficiency. For example, lack of netrin-1 causes flattening of the mDA neuron pool which could impede the rostral migration of *Pax7*^*+*^ neurons into the IPN.^43^^,^^76^^,^^77^ Vice versa, failed *Pax7*^*+*^ neuron migration may impact the mDA neuron pool. In all, these data show a subtype-specific role for netrin-1 in the migration and positioning of GABAergic neurons in the ventral midbrain. This function is highly regulated with respect to the origin of netrin-1 and netrin-1 receptor involvement and adds further complexity to the role of netrin-1 in ventral midbrain development, i.e., in addition to reported functions in mDA neuron migration,^28^^,^^50^ fasciculus retroflexus bundling,^78^^,^^79^^,^^80^ and synaptic signaling.^81^ Further, netrin-1 has been associated with several psychiatric and neurodegenerative disorders^82^^,^^83^^,^^84^ and our data implicating this cue in GABAergic neuron subtype development provide a potential avenue for further comprehending underlying pathogenic mechanisms and for developing therapeutic interventions.

In conclusion, our work reveals an unexpected level of molecular heterogeneity in GABAergic neuron populations flanking the mDA neuron pool and provides a molecular starting point for the further dissection of the mechanisms underlying the development and function of ventral midbrain GABAergic subtypes in the healthy and diseased brain.

### Limitations of the study

One limitation of a FACS-based strategy as used in our study is that it includes cells that at any moment up to the time point of analysis express *VGAT* and not cells that perse express *VGAT* at the selected time point. However, the expression of *Slc32a1* in all scRNA-seq clusters suggests that in the current study this is not a major confounding factor.

Further, while our study highlights developmental aspects of GABAergic neurons, an important future goal is to investigate whether the GABAergic subpopulations identified here maintain their identity toward adult stages. Transcript levels can fluctuate over time and early developmental stages often feature high expression of specific markers that help to define and distinguish subclusters. However, this expression may decrease as cells progress toward subsequent developmental stages. For example, vGlut2 is broadly expressed in mDA neurons during development but becomes more restricted in adulthood.^85^ Future studies are needed to determine whether VGAT expression exhibits a similar temporal expression profile.

## Resource availability

### Lead contact

Further information and requests for resources and reagents should be directed to and will be fulfilled by the lead contact, Jeroen Pasterkamp (R.J.Pasterkamp@umcutrecht.nl).

### Materials availability

All unique reagents, plasmids, and transgenic mouse lines generated in this study are available from the lead contact with a completed Materials Transfer Agreement.

### Data and code availability

• Data: scRNA-seq data generated in this paper are deposited at GEO database (GEO: GSE268378) and are publicly available. This information is listed in the key resources table.
• Code: This paper does not report original code.
• Other: Any additional information required to reanalyze the data reported in this paper is available from the lead contact upon reasonable request.

## Acknowledgments

We thank Christiaan van der Meer and Anna de Ruiter for technical assistance; Robert Burgess, Lisa Goodrich, Anton Berns, Marten Smidt, Mario Capecchi, Alain Chedotal, Jean-Francois Cloutier, Alessandra Pierani, and Corette Wierenga for mouse lines and tissues; Rudolf Jaenisch for *PCAGS-FLPe-puro*; and Juha Partanen for cDNAs for ISH probes. This research was supported by the 10.13039/501100003246Netherlands Organisation for Scientific Research (ALW-VICI 865.14.004) and 10.13039/501100008383Stichting ParkinsonFonds (to R.J.P.). Partially supported by NWO Gravitation program BRAINSCAPES: Roadmap from Neurogenetics to Neurobiology (NWO: 024.004.012) (to R.J.P.).

## Author contributions

Ö.D., D.D.A.R., and R.J.P. designed the study; Ö.D. and R.J.P. wrote the manuscript with help from all authors; Ö.D. and D.D.A.R. designed and performed experiments with help from L.L.vd.H., L.M.G. L.E.L., O.G., Y.A., N.C.H.v.K., M.H.B., and T.H.W.K.; F.J.M. designed experiments and aided in data analysis; supervision and funding acquisition by R.J.P.

## Declaration of interests

The authors declare no competing interests.

## STAR★Methods

### Key resources table

**Table:** 

REAGENT or RESOURCE	SOURCE	IDENTIFIER
**Antibodies**		

rabbit anti-TH	Millipore	Cat# AB152 RRID: AB_390204
sheep anti-TH	Millipore	Cat# AB1542; RRID: AB_90755
mouse anti-TH	Millipore	Cat# MAB318; RRID: AB_2201528
chicken anti-TH	Aves labs	Cat#: TYH; RRID: AB_10013440
rabbit anti-Six3	Rockland	Cat# 600-401-A26; RRID: AB_11180063
rabbit anti-Pax5	Abcam	Cat# ab109443; RRID: AB_10862070
goat anti-Sox21	R&D Systems	Cat# AF3538; RRID: AB_2195947
rat anti-CTIP2	Abcam	Cat# AB18465; RRID: AB_2064130
rabbit anti-Pou6f2	Sigma-Aldrich	Cat# HPA008699; RRID: AB_1079664
goat anti-Otx2	R&D Systems	Cat# Af1979; RRID: AB_2157172
mouse anti-En1	DSHB	Cat#4G11; RRID: AB_528219
mouse anti-Nkx2.2	DSHB	Cat#74.5A5; RRID: AB_531794
mouse anti-Pax7	DSHB	DSHB, Cat#pax7; RRID: AB_528428
goat anti-tdTomato	SICGEN	Cat#AB8181; RRID: AB_2722750
rabbit anti-RFP	Rockland	Cat#600-401-379; RRID: AB_2209751
rabbit anti-GFP	Life technologies	Cat#A11122; RRID: AB_221569
anti-DIG-AP	Sigma	Cat#11207733910; RRID: AB_2734716
donkey anti-rabbit Alexa Fluor 488	Invitrogen	Cat#A21206; RRID: AB_2535792
donkey anti-sheep Alexa Fluor 488	Invitrogen	Cat#A11015; RRID: AB_2534082
donkey anti-mouse Alexa Fluor 488	Invitrogen	Cat#A21202; RRID:AB_141607
donkey anti-rabbit Alexa Fluor 568	Abcam	Cat#ab175470; RRID: AB_2783823
donkey anti-goat Alexa Fluor 568	Abcam	Cat#ab175474; RRID: AB_2636995
donkey anti-mouse Alexa Fluor 568	Invitrogen	Cat#A10037; RRID: AB_11180865
donkey anti-rabbit Alexa Fluor 647	Invitrogen	Cat#A31573; RRID: AB_2536183
donkey anti-rat Alexa Fluor 647	Abcam	Cat#ab150155; RRID: AB_2813835
donkey anti-mouse Alexa Fluor 647	Invitrogen	Cat#A32787; RRID: AB_2762830
donkey anti-goat IgG Alexa Fluor 647	Abcam	Cat#ab150135; RRID: AB_2687955
donkey anti-rabbit IgG H&L Alexa Fluor 750	Abcam	Abcam Cat# ab175731; RRID: AB_2943056

**Chemicals, peptides, and recombinant proteins**		

HBSS – Ca2+ - Mg2+	Thermo-Fisher	Cat#24020117
D-Trehalose Dihydrate	Alfa Aesar	Cat#A19434
Bovine Serum Albumine	Jackson	Cat#001-00-162
HEPES	Thermo-Fisher	Cat#15630106
Methanol	Merck Millipore	Cat#1060092500
Hydrogen peroxide	Merck Millipore	Cat#1072091000
Triton X-100	Sigma	Cat#x100-500mL
Thimerosal	Gerbu	Cat#1031/USP35
Saponin	Sigma	Cat#S7900
Tetrahydroflurane	Sigma	Cat#186562-1L
Dichloromethane	Sigma	Cat#270997-1L
Dibenzyl ether	Sigma	Cat#108014-1kg
Heparin	Sigma	Cat#H3393
Formaldehyde	Riedel-de Hain	Cat#33200
Glutaraldehyde	Acros Organics	Cat#119989925
Sodium deoxycholate	Sigma	Cat#D6750-100G
K-ferricyanide	Merck	Cat#231-847-6
K-ferrocyanide	Sigma	Cat#p8131-100G
Magnesium Chloride	Merck	Cat#A0344733339
DNase	Roche	Cat#776785
EDTA	Sigma	Cat#E5134-500G
Lithium Chloride	Merck	Cat#7447-41-8
Triethanolamine	Fluka	Cat#90279
Acetic anhydride	Sigma	Cat#A6404
Deionized formamide	ICN	Cat#800686
Ficoll-400	Sigma	Cat#F4375
Polyvinylpyrrolidone	Merck	Cat#7443
BSA-fraction V	ICN	Cat#103703
tRNA baker’s yeast	Sigma	Cat#R-6750
SonifiCated Salmon Sperm DNA	Sigma	Cat#D-91565
NBT/BCIP	Boehringer Mannheim	Cat#1681451
Levamisol	Sigma	Cat#L9756
Normal Donkey Serum	Jackson Immunoresearch	Cat#017-000-121
Fluorsave	VWR international	Cat# 345789-20P
DAPI - 4′,6-diamidino-2-phenylindole	Sigma	Cat#D9564
Papain	Worthington Biochemical	Cat#LK003178
DNase I type IV	Sigma	Cat#D5025
L-cysteine	Sigma-Aldrich	Cat#C7352
NaHCO3	Sigma-Aldrich	Cat#S5761
FluorSave	VWR international	Cat#345789-20
ProLong Gold antifade mountant	Invitrogen	Cat#P36984

**Critical commercial assays**		

PureLink Quick Gel Extraction kit	Thermo-Fisher	Cat#K210025

**Deposited data**		

scRNA-seq data (FastQ and count table for GABAergic neurons in the ventral midbrain)	this paper	GEO: GSE268378

**Experimental models: Organisms/strains**		

Mouse: C57BL/6j	Charles River	Cat#027; RRID:IMSR_JAX:000664
Mouse: STOP-tdTomato (Ai14)	The Jackson Laboratory	JAX stock #007914, RRID:IMSR_JAX:007914
Mouse: Ai65F	The Jackson Laboratory	JAX stock #032864, RRID:IMSR_JAX:032864
Mouse: RCE:FRT	The Jackson Laboratory	JAX stock #032038, RRID: MMRRC_032038
Mouse: VGAT-Cre	The Jackson Laboratory	JAX stock #016962, RRID:IMSR_JAX:016962
Mouse: DSCAM−/−	The Jackson Laboratory	JAX stock # 006038, RRID:IMSR_JAX:006038
Mouse: FoxG1-Cre	The Jackson Laboratory	JAX stock #029690, RRID:IMSR_JAX:029690
Mouse: Tag1-Cre	Brignani et al.^28^	N/A
Mouse: netrin-1−/−	gift of Lisa Goodrich (Harvard Medical School)	N/A
Mouse: netrin-1ffl/f	gift of Lisa Goodrich (Harvard Medical School)	N/A
Mouse: DCCfl/fl	gift from Anton Berns (Netherlands Cancer Institute)	N/A
Mouse: Dbx1-Cre	Alessandra Pierani (INSERM)	N/A
Mouse: EIIa-Cre	The Jackson Laboratory	JAX stock # 003724, RRID:IMSR_JAX:003724
Mouse: DCC−/−	Generated by crossing DCCfl/fl and EIIa-Cre	N/A
Mouse: Pitx3-Cre	gift from Marten Smidt (University of Amsterdam)^86^	N/A
Mouse: Pitx3-GFP	gift from Marten Smidt (University of Amsterdam)^86^	N/A
Mouse: HoxB1-Cre	gift from Mario Capecchi (University of Utah)	N/A
Mouse: DTA	The Jackson Laboratory	JAX stock # 009669; RRID:IMSR_JAX:009669
Mouse brain: En1-Cre:netrin-1fL/fL	provided by Alain Chédotal^53^	N/A
Mouse: Pitx3-FlpE	this paper	N/A

**Oligonucleotides**		

See Table S5		

**Software and algorithms**		

Scanpy	Theis lab SCANPY: large-scale single-cell gene expression data analysis F. Alexander Wolf, Philipp Angerer, Fabian J. Theis Genome Biology 2018 Feb 06. https://doi.org/10.1186/s13059-017-1382-0.	https://github.com/theislab/scanpy; RRID:SCR_018139
MapAndGo2 STARmap	Anna Alemany	https://github.com/anna-alemany/transcriptomics/tree/master/mapandgo
Python Software Foundation	Python Software Foundation	RRID:SCR_008394; https://www.python.org/
Louvain	vtraag/louvain-igraph: 0.6.1	https://doi.org/10.5281/zenodo.595481
Graphpad Prism version 9.1.1	GraphPad Software	RRID:SCR_002798; https://www.graphpad.com/
PatchMaster v2x90.2	HEKA Elektronik GmbH	N/A
Imaris software (version >9.4, Bitplane)	Bitplane	RRID:SCR_007370; http://www.bitplane.com/imaris/imaris
Imspector software version 7.6.3.	LaVision BioTec	N/A
Zen 2 (blue edition)	Zeiss	N/A
ImageJ version 1.54f	ImageJ	RRID:SCR_003070
Cell Counter version 3.0.0	ImageJ	N/A

### Experimental model and subject details

#### Animals

All mice used in this study were housed socially and kept under a normal 12 h:12 h light-dark cycle. Mice were kept at room temperature (RT; 22 ± 1°C) and were fed with chow and water *ad libitum*. All mouse experiments were approved by the Animal Ethics Committee of Utrecht University (Dierexperimenten Ethische Commissie) (CCD license: AVD115002016532 until June 2021; AVD11500202114777 after June 2021) and conducted in agreement with Dutch law (Wet op de Dierproeven, 1996; revised 2014) and European regulations (Guideline 86/609/EEC; Directive 2010/63/EU). The sex of embryos was not considered in this study.

The following mice were used: C57bl6J (Charles Rivers Laboratories), *STOP-tdTomato (Ai14)* (JAX stock #007914, RRID:IMSR_JAX:007914),^87^ *Ai65F* (JAX stock #032864, RRID:IMSR_JAX:032864), *RCE:FRT* (JAX stock #032038, RRID: MMRRC_032038), *VGAT-Cre* (JAX stock #016962, RRID:IMSR_JAX:016962),^88^ *DSCAM*^*−/−*^ (JAX stock # 006038, RRID:IMSR_JAX:006038),^89^ *FoxG1-Cre* (JAX stock #029690, RRID:IMSR_JAX:029690),^90^ and *Tag1-Cre*.^28^ *netrin-1*^*−/−*^ and *netrin-1*^*ffl/fl*^ mice were a kind gift of Lisa Goodrich (Harvard Medical School).^91^ *DCC*^*fl/fl*^ mice were a kind gift from Anton Berns (Netherlands Cancer Institute).^92^ *Dbx1-Cre* mice were a kind gift from Alessandra Pierani (INSERM). *DCC*^*−/−*^ mice were generated by crossing *DCC*^*fl/fl*^ and *EIIa-Cre* (JAX stock # 003724, RRID:IMSR_JAX:003724) mice. *Pitx3-Cre* and *Pitx3-GFP* mice were a kind gift from Marten Smidt (University of Amsterdam).^86^ *HoxB1-Cre* mice were a kind gift from Mario Capecchi (University of Utah).^93^ *En1-Cre:netrin-1*^*f*^^*l*^^*/f*^^*l*^ and control brains were provided by Alain Chedotal.^53^ Genotyping was performed as described previously (Table S5).^28^

#### Generation of Pitx3-FlpE mice

*Pitx3-FlpE* mice were generated by the ETH Phenomics Center (EPIC) of ETH Zürich (Switzerland) using a similar strategy as used for the generation of *Pitx3-Cre* mice.^86^ In short, *Pitx3* BAC *RP23-125F3* (BACPAC Resources Center at Children’s Hospital Oakland Research Institute (CHORI)) was used as a template for the generation of homologous sequences resulting in the substitution of *Pitx3* exons 2–4 by FlpE. The FlpE sequence was amplified from *PCAGS-FLPe-puro*^94^ (Addgene, #20733), which was a kind gift from Rudolf Jaenisch. PCR fragments containing Pitx3 homology arms, FlpE recombinase containing *loxP* sites, splice acceptors, and a poly-A tail were inserted into the cloning vector pBS-KS in a multi-step cloning process. The final targeting vector, 17004.5a TV, was verified by restriction digestion and sequencing, and electroporated into *C57bl/6N* based ESCs. Positive clones were identified by Southern blot analysis and microinjected into blastocytes. *Pitx3-FlpE* mice were genotyped by PCR (Table S5).

### Method details

#### Immunohistochemistry and image quantification

To collect embryos, pregnant mice were sacrificed by cervical dislocation. The morning on which a vaginal plug was observed was considered embryonic (E) day 0.5. Embryos were harvested at E16.5, E18.5 and postnatal (P) day 0.5 and placed in ice-cold 1× PBS. Both postnatal and embryonic brains were isolated and fixed overnight (ON) at 4°C in 4% PFA prepared in PBS. Brains were cryoprotected in 30% sucrose prepared in 1× PBS for 24 h. After the brains sunk, they were frozen in isopentane and stored at −80°C. Brain sections of 25 μm thickness were generated on a cryostat. First, sections were blocked with blocking solution (0.4% Triton X-100 and 1% bovine serum albumin (BSA), in 1× PBS) for 1 h at RT. Primary antibodies diluted in blocking solution were added to the sections and incubated ON at 4°C. The following primary antibodies were used: rabbit anti-TH 1:500 (Millipore, Cat# AB152, RRID: AB_390204), sheep anti-TH 1:500 (Millipore, Cat# AB1542, RRID: AB_90755), mouse anti-TH 1:500 (Millipore, Cat# MAB318, RRID: AB_2201528), chicken anti-TH (Aves labs, Cat#TYH, RRID: AB_10013440), rabbit anti-Six3 1:1000 (Rockland, Cat# 600-401-A26, RRID: AB_11180063), rabbit anti-Pax5 1:500 (Abcam, Cat# ab109443; RRID: AB_10862070), goat anti-Sox21 1:200 (R&D Systems, Cat# AF3538, RRID: AB_2195947), rat anti-CTIP2 1:1000 (Abcam, Cat# AB18465, RRID: AB_2064130), rabbit anti-Pou6f2 1:500 (Sigma-Aldrich, Cat# HPA008699, RRID: AB_1079664), goat anti-Otx2 1:500 (R&D Systems, Cat# Af1979, RRID: AB_2157172), mouse anti-En1 1:40 (DSHB, Cat#4G11, RRID: AB_528219), mouse anti-Nkx2.2 1:10 (DSHB, Cat#74.5A5, RRID: AB_531794), mouse anti-Pax7 1:50 (DSHB, Cat#pax7, RRID: AB_528428), goat anti-tdTomato 1:500 (SICGEN, Cat#AB8181, RRID: AB_2722750), rabbit anti-RFP 1:500 (Rockland, Cat#600-401-379, RRID: AB_2209751), rabbit anti-GFP 1:2000 (Life technologies, Cat#A11122, RRID: AB_221569). Sections were washed with 1× PBS and subsequently incubated with secondary antibodies (1:750) in 1× PBS for 1 h at RT. The following secondary antibodies were used: donkey anti-rabbit Alexa Fluor 488 (Invitrogen, Cat#A21206, RRID: AB_2535792), donkey anti-sheep Alexa Fluor 488 (Invitrogen, Cat#A11015, RRID: AB_2534082), donkey anti-mouse Alexa Fluor 488 (Invitrogen, Cat#A21202, RRID: AB_141607), donkey anti-rabbit Alexa Fluor 568 (Abcam, Cat#ab175470, RRID: AB_2783823), donkey anti-goat Alexa Fluor 568 (Abcam, Cat#ab175474, RRID: AB_2636995), donkey anti-mouse Alexa Fluor 568 (Invitrogen, Cat#A21202, RRID: AB_11180865), donkey anti-rabbit Alexa Fluor 647 (Invitrogen, Cat#A31573, RRID: AB_2536183), donkey anti-rat Alexa Fluor 647 (Abcam, Cat#ab175750, RRID: AB_2813835), and donkey anti-mouse Alexa Fluor 647 (Invitrogen, Cat#A21202, RRID: AB_2762830). Next, sections were washed with 1× PBS and counterstained with DAPI (4′,6′-diamidino-2-phenylindole; 0.1 mg/mL in 1× PBS, Invitrogen). Slides were mounted with FluorSave reagent (VWR international, Cat#345789-20) or ProLong Gold antifade mountant (Invitrogen, Cat# P36984). Images were obtained using an Axioscope A1 fluorescence microscope (Zeiss) or by confocal laser-scanning microscopy (LSM880, Zeiss).

Cell counts were obtained using the Cell Counter ImageJ plugin. En1^+^ and Otx2^+^ cells (Figure 4I) were quantified in both sides of the lateral VTA (*n* = 4, 2 different levels on the rostral-caudal axis on both sides of the DA system when staining allowed). TH^+^ and tdT^+^ cells (Figures S5C and S5D) were quantified in the mDA neuron pool (*n* = 3, 3 different levels on the rostral-caudal axis).

#### *In situ* hybridization

##### Probe generation

Probes were generated as described previously.^95^ In brief, primers were designed using the primer designing tool from NCBI (link) or taken from the Allen Brain atlas. A two-step PCR was performed. First probe sequences were amplified from whole brain mouse cDNA. cDNA for *En1*, *Tal1*, *Six3* and *Gad1* were a kind gift from Juha Partanen (University of Helsinki) (Table S5). For the second PCR, the purified product of the first PCR or cDNAs were used as a template. Primers for the second PCR contained a T7 overhang for antisense and T3 overhang for sense probe generation. After purification of the second PCR product, *in vitro* transcription was performed to synthesize digoxigenin (DIG)-labeled antisense RNA probes using T7 RNA polymerase and sense RNA probes using T3 RNA polymerase (Table S5). No specific signals were detected in *in situ* hybridization experiments in which sense probes were used.

##### *In situ* hybridization

Nonradioactive *in situ* hybridization was performed as described previously.^28^ In brief, PFA-fixed sections were dried 2 h at RT and washed 3 × 5 min in 1x PBS containing 1% Tween 20. Sections were permeabilized with 5 μg/mL proteinase K in 1x PBS for 5 min and post-fixed in 4% PFA for 10 min. Slides were washed 3 × 3 min in 1× PBS. To prevent aspecific binding of RNA probes, sections were acetylated (0.25% acetic anhydride in 0.1 M triethanolamine and 0.06% HCl) for 10 min at RT. Hybridization solution (5× saline sodium citrate (SSC; 0.75 M NaCl, 75 mM sodium citrate, pH 7.0), 5× Denhardts, 50% deionized formamide, 250 mg/mL tRNA baker’s yeast, 500 mg/mL sonicated salmon sperm DNA) was added to the sections for 2 h at RT. Next, 0.5–2 μg of DIG-labeled RNA probe was diluted in the hybridization mix and added to the sections. Slides were covered with NESCO-film and placed in a humidified chamber at 68°C ON. The next day, slides were placed in 2× SSC and washed in 0.2× SSC for 2 h at 68°C. Samples were washed again in 0.2× SSC for 5 min at RT and with Buffer 1 (100 mM Tris hydrochloride (HCl), pH 7.4) for another 5 min. Sections were blocked with Buffer 1 containing 10% heat inactivated FCS for 1 h at RT and incubated with anti-DIG AP antibody (1:5000 in Buffer 1 containing 1% FCS) in combination with other primary antibodies ON at 4°C. The next day, sections were washed 3 × 5 min in Buffer 1 on a shaker. Secondary antibodies were added for 1 h at RT and washed 3 × 5 min in Buffer 1 and another 5 min in Buffer 2 (100 mM Tris HCl, 50 mM MgCl_2_, 100 mM NaCl, pH 9.5). For the color reaction, BCIP/NBT (1:20 ratio) and 0.24 mg/mL Levamisole were diluted in Buffer 2 and added to the sections for 2- 24 h. Sections were mounted in ProLong Gold antifade reagent or FluorSave. Images were obtained using a digital slide scanner (Hamamatsu Nanozoomer), using a Zeiss Axioscope A1 fluorescence microscope (Zeiss) or by confocal laser-scanning microscopy (LSM 880, Zeiss).

#### Tissue clearing and fluorescent light sheet microscopy

P5 *VGAT-Cre:Pitx3-FlpE:Ai65* mouse brains were cleared and stained using the iDISCO protocol as described previously.^96^^,^^97^ In short, perfused, isolated, and post-fixed brains were gradually dehydrated in a series of 20% MeOH, 40% MeOH, 60% MeOH, 80% MeOH, and 100% MeOH (2×), for 1 h at RT each, followed by ON incubation at RT in 66% DCM and 33% MeOH. The next day, samples were incubated in 100% MeOH (2×) for 1 h at RT, followed by incubation at 4°C > 1 h (to cool the sample for bleaching). Next, samples were bleached ON in 5% hydrogen peroxide in MeOH at 4°C. The following day, samples were gradually rehydrated using a series of 80% MeOH, 60% MeOH, 40% MeOH, 20% MeOH for 1 h each at RT. Samples were then washed with 1× PBS for 1 h at RT. Subsequently, samples were incubated twice in Ptx.2 (1× PBS and 0.2% Triton X-100) for 1 h at RT and transferred into permeabilization solution (80% Ptx.2, 20% DMSO, 2.3% glycine) for 24 h at 37°C on a horizontal shaker (70 rpm). This shaker was also used for blocking and antibody incubations with the same settings. Samples were incubated in blocking solution for 4 days, followed by incubation in primary antibody solution (PwtH (1× PBS, 0.2% Triton X-100, 0.1% Heparin (Sigma, Cat#H3393, 10 mg/mL)) stock solution, 5% DMSO and 3% normal donkey serum (NDS) (Jackson Immunoresearch, Cat#017-000-121)) for 7 days at 37°C while shaking. Then, samples were washed 5 times for 1 h at RT in a 15 mL Falcon tube containing PtwH. Samples were incubated in secondary antibody solution (PwtH and 3% NDS (Jackson Immunoresearch, Cat#017-000-121)) for 7 days at 37°C on a horizontal shaker (70 rpm) in a 5 mL Eppendorf.

Primary antibodies used were goat anti-tdTomato 1:500 (SICGEN, Cat#AB8181, RRID: AB_2722750) in combination with secondary donkey anti-goat IgG Alexa Fluor 647 1:750 (Abcam, Cat#ab150135; RRID: AB_2687955), and rabbit anti-TH 1:500 (Millipore, Cat# AB152, RRID: AB_390204) with secondary donkey anti-rabbit IgG H&L Alexa Fluor 750 1:750 (Abcam Cat# ab175731, RRID: AB_2943056). Then, samples were dehydrated using a series of 20% MeOH, 40% MeOH, 60% MeOH, 80% MeOH and 2 times 100% MeOH, each step for 1 h at RT. Samples then were placed in 66% DCM and 33% MeOH for 3 h at RT, followed by 2 washes in 100% DCM for 15 min at RT each. After removing lipids with Dichlormethan (DCM), clearing was finalized by incubation in Dibenzylether (DBE). For clearing, tissue samples were placed in 100% DBE overnight at RT. Samples were stored in 100% DBE at RT until imaging. All washing, dehydration and clearing steps were performed in dark Falcon tubes to protect against light and on a rotator (14 rpm).

Samples were imaged in horizontal orientation using an UltraMicroscope Blaze (Miltenyi Biotec) light sheet microscope equipped with a tube lens magnification change unit (0.6×, 1×, 1.67× and 2.5×), and MI Plan objective lenses, 4.2 Megapixel sCMOS camera (2048 × 2048 pixels, pixel size: 6.5 × 6.5 μm2) and Imspector software (7.6.3.). Samples were scanned using single-sided illumination, a sheet NA of 0.108 and a step-size of 2.5 μm using the horizontal focusing light sheet scanning method with 11 steps (optimal) and using the blend algorithm. For all images shown, a 4× objective was used (MI Plan 4×/0.35 NA). The effective magnification was 2.4× (tube lens magnification ∗ objective = 0.6× ∗ 4×). Imaris software (version >9.4, Bitplane) was used for image processing and analysis.

#### Electrophysiology

*VGAT-Cre:Ai14:Pitx3-GFP* mice were sacrificed between postnatal day 3 and 12. Coronal brain slices of 250 μm were cut on a vibratome (1200 VTs, Leica, Rijswijk, The Netherlands) in ice-cold carbonated (95% O_2_, 5% CO_2_) cutting solution, containing (in mM) choline chloride 92; ascorbic acid 10; CaCl_2_ 0.5; glucose 25; HEPES 20; KCl 2.5; N-Acetyl L Cysteine 3.1; NaHCO_3_ 25; NaH_2_PO4 1.2; NMDG 29; MgCl_2_ 7; sodium pyruvate 3; Thiourea 2. Slices were transferred for 5 min to warmed solution (34°C) of identical composition, before they were stored at RT in carbonated incubation medium containing (in mM) ascorbic acid 3; CaCl_2_ 2; glucose 25; HEPES 20; KCl 2.5; NaCl 92; NaHCO_3_ 20; NaH_2_PO_4_ 1.2; NMDG 29; MgCl_2_ 2; sodium pyruvate 3 and Thiourea 2. During recordings, slices were immersed in artificial cerebrospinal fluid (ACSF) containing (in mM) CaCl_2_ 2.5; glucose 11; HEPES 5; KCl 2.5; NaCl 124; NaHCO_3_ 26; NaH_2_PO_4_ 1; MgCl_2_ 1.3 and were continuously superfused at a flow rate of 2.5 mL min^−1^ at 32°C.

Neurons were patch-clamped using borosilicate glass pipettes (2.7–4 MΩ; glass capillaries, GC150-10, Harvard apparatus, UK), under a TH4-200 Olympus microscope (Olympus, France). For voltage or current clamp recordings, signal was amplified, low-pass filtered at 2.9 kHz with a 4-pole Bessel filter, and digitized at 20 kHz with an EPC10 dual patch-clamp USB amplifier (HEKA Elektronik GmbH). Data were acquired using PatchMaster v2x90.2software. Patch clamp recordings were made in solution containing (in mM), Potassium Gluconate 139; HEPES 10; EGTA 0.2; creatine phosphate 10; KCl 5; Na_2_ATP 4; Na_3_GTP 0.3; MgCl_2_ 2. Intrinsic excitability recordings were made in current clamp as before.^98^ To assess the membrane resistance, firing pattern, action potential threshold and voltage sag, neurons were subjected to current steps of 800 ms length, starting from −100 pA, with a 25 pA increasing increment/step, and 10 s inter-sweep intervals. Cellular capacitance was calculated by applying a −4 mV step in voltage clamp to determine the membrane time constant tau by exponential curve fitting of the current recovery and dividing it by the series resistance. A Patch map was based on the position of recorded neurons post hoc, scored blindly with respect to cell type, and drawn into a Paxinos schematic of the midbrain.

#### Tissue collection and FACS

The brains of E16.5 and P0.5 *VGAT-Cre:Ai14:Pitx3-GFP* mice were isolated and dissected in medium-A (HBSS containing 0.6% D-Glucose, 15 mM HEPES buffer and 50% D-Trehalose Dihydrate (Alfa Aesar)) on ice. In total, 14 brains from 6 litters of E16 embryos and 13 brains from 6 litters of P0 pups were used for scRNA-seq analysis. The meninges was removed and brains were cut in 300 μm slices using a tissue chopper. The ventral midbrain was cut out with a knife and collected into a tube. Tissue pieces were dissociated by papain treatment (12 U/mL papain (Worthington Biochemical); 250 U/mL DNase I type IV (Sigma); 3.5 mM L-cysteine (Sigma-Aldrich); 0.215% NaHCO_3_ (Sigma-Aldrich); 5 mM EDTA (Life Technologies); 0.2% Phenol Red (Sigma-Aldrich); 1 mM sodium pyruvate (Life Technologies); 1.8 mg/mL D-Trehalose Dihydrate (Alfa Aesar); 50 U/mL penicillin and 50 mg/mL streptomycin (Life Technologies) in HBSS without Ca^2+^and Mg^+^ (Life Technologies)). After aspiration of papain, tissue was mechanically dissociated using fire polished Pasteur pipettes in Trituration solution (0.2% BSA (Sigma-Aldrich); 50 U/mL penicillin, 50 mg/mL streptomycin; 1 mM sodium pyruvate; 1.8 mg/mL glucose; 250 U/mL DNase I type IV in Neurobasal medium (Life Technologies)). Dissociated cells were purified on BSA columns (1.8% (w/v) BSA; 50 U/mL penicillin; 50 mg/mL streptomycin; 1 mM sodium pyruvate; 1.8 mg/mL glucose; 250 U/mL DNase I type IV; 3 mM NaOH (Fisher Scientific) in Neurobasal medium) and separated from debris by centrifugation at 300 *g* for 5 min. Cells were resuspended in medium-A and filtered through a 70 μm filter into a FACS tube. Five minutes prior to sorting, 3 μL of 1 μg/mL DAPI solution was added to the cells. Cells were sorted and collected into 384 well plates in a BD FACSAria II Flow Cytometer (BD Biosciences) with a 100 μm nozzle. Plates were subsequently centrifuged for 1 min at 1000 rpm and stored at −80°C. Nine plates were collected and VGAT^+^Pitx3^+^ cells were collected to fill one plate for each developmental stage (Figure S1). Therefore, while VGAT^+^Pitx3^+^ cells normally represent approximately 2% of all VGAT^+^ cells, they are overrepresented in the study due to more extensive sampling to aid their analysis.

#### Single-cell RNA sequencing

Single-cell RNA sequencing (scRNA-seq) was performed at Single Cell Discoveries (Utrecht, The Netherlands) using the SORT-seq technique, based on CEL-Seq2.^99^ In brief, cells were lysed, after which the poly-T stretch of the barcoded primers were hybridized to the poly-A tail of the mRNA molecules. Primers consisted of a 24 bp poly-T stretch, a 4 bp random molecular barcode (UMI), a cell-specific 8 bp barcode, the 5′ Illumina TruSeq small RNA kit adapter and a T7 promoter. DNA-RNA hybrids were then reverse transcribed, converted to cDNA, containing the mRNA and the primer sequence and subsequently converted into second stranded DNA by a DNA polymerase I step. The cDNA was pooled and *in vitro* transcribed for amplification. Next, the amplified RNA was reverse transcribed using random hexamer primers. Illumina sequencing libraries were prepared with TruSeq small RNA primers (Illumina) and sequenced paired-end at 75 bp read length using Illumina NextSeq.

The mapping process was carried out utilizing the MapAndGo255 STARmap pipeline. Briefly, fastq files from different lanes were merged to consolidate the data, followed by the removal of reads without a CEL-Seq2 barcode during the demultiplexing step. To enhance data quality, Illumina adapter sequences and low-quality base calls from the 3′ end of the reads were trimmed. The subsequent step involved aligning the reads to a reference mouse genome (Ensemble GRC38 release 93) using STAR v-2.5.3a. This alignment procedure generated three distinct count files: the unspliced file containing reads mapped to intronic regions, the spliced file containing reads mapped to exons, and a file comprising both types of reads. These three files were then utilized as input for the SCANPY pipeline.

##### scRNA-seq data filtering, normalization and analysis

Data analysis was conducted using Python (version 3.7.6) and the SCANPY package (version 1.4.6.).^100^ The *VGAT-Cre:Ai14* dataset consisted of 3,455 cells, expressing a total of 32,787 genes. Among the reads, 82% were mapped to exonic regions, while 18% were mapped to intronic regions. The median read depth per cell was 7,260 reads, and the median number of genes detected per cell was 3,219. To assess sequencing quality, External RNA Control Consortium (ERCC) spike-ins, which are synthetic RNA molecules, were added to each cell.^101^ By examining the number of ERCC reads per cell, the quality of sequencing could be determined. The dataset exhibited a median sequencing quality of 0.04% ERCC spike-ins per cell. For further processing, cell filtering was performed, and ERCC genes along with mitochondrial genes were removed. Cells expressing a range of 2,000–50,000 genes were retained, with less than 15% mitochondrial genes, 20% ribosomal genes, and 30% ERCC spike-ins. Additionally, at least 85% of the genes had to be mapped to exonic regions. In addition to cell filtering, gene filtering was also applied to select genes with the highest fraction of counts within each cell, as well as genes expressed in more than 3 cells. After filtering, the dataset contained 2,042 cells, expressing a total of 25,122 genes. The median read depth per cell was 14,468 reads, and the median number of genes detected per cell was 5,070. The raw gene expression matrix was normalized to 10,000 reads per cell and log-transformed. Genes with expression levels ranging from 0.0125 to 3 and a minimum dispersion of 0.5 were considered highly variable and retained for clustering analysis. To account for the effects of total counts per cell and the percentage of mitochondrial reads, regression was performed, and data were scaled to a maximum value of 10.

Principal Component Analysis (PCA) was conducted, considering fifty components. The nearest neighbors were then determined based on the first forty principal components. Subsequently, t-SNE coordinates were computed utilizing the first forty principal components. For clustering analysis, the Louvain algorithm^102^ was used. To determine marker genes for each Louvain cluster, the Wilcoxon rank-sum test was employed using the sc.tl.rank_genes_groups() function in SCANPY. To identify more specific marker genes, the marker genes were filtered based on the following criteria: a minimum fraction of occurrence within the group of 0.5, a maximum fraction of occurrence outside the group of 0.3, and a minimum fold change of 1.5. These ranked gene groups were then used to annotate cluster groups by determining whether marker genes had previously been described as marker genes for specific regions or cell types in the ventral midbrain using literature searches. Subsequently their spatial expression was checked at E18 and P0 using the Allen Brain Atlas (ABA) to confirm this anatomical location. If no ABA data were available additional immunostainings or *in situ* hybridizations were performed.

### Quantification and statistical analysis

Correlational analysis was performed to evaluate the relationship between postnatal age (P3-P12) and electrophysiological properties. Parameters assessed include the number of action potentials (APs) fired spontaneously at 0 pA, and in response to 100 pA and 200 pA inputs, along with resting membrane potential, membrane resistance, action potential threshold, cellular capacitance, and voltage sag (elicited by a −100 pA step). Pearson’s correlation analysis was used, and unadjusted *p*-values were reported. Significance was considered at both unadjusted (α = 0.05) and adjusted (α = 0.00625) levels. Bonferroni correction was applied for eight comparisons within each cell type.

All statistical analyses were conducted using GraphPad Prism version 9.1.1. two-Way ANOVA with repeated measures was used for comparisons between cell types, followed by Tukey post hoc tests. For main effects of cell type, One-Way ANOVA was employed. Sample size for each experiment is indicated in the figure legends. All statistical details of experiments can be found in the figure legends.

For quantification, data are presented as means ± SEM. The number of En1^+^/Otx2^+^ and Otx2^+^/En1^+^ cells was analyzed using the Shapiro-Wilk test for normality, followed by two-tailed t-tests (∗∗∗∗*p* < 0.0001).
