# Supplementary material for: Molecular diversity and migration of GABAergic neurons in the developing ventral midbrain
Source: iScience. 2024 Oct 23;27(11):111239. doi: 10.1016/j.isci.2024.111239 (PMC11576407; doi:10.1016/j.isci.2024.111239)
Supplement: Document S1. Figures S1–S6 and Table S1–S5 [file mmc1.pdf]

iScience, Volume *27*

Supplemental information

Molecular diversity and migration of GABAergic neurons in the developing ventral midbrain

Özge Düdükcü, Divya D.A. Raj, Lieke L. van de Haar, Laurens M. Grossouw, Louisa E. Linders, Oxana Garritsen, Youri Adolfs, Nicky C.H. van Kronenburg, Mark H. Broekhoven, Troy H.W. Kapteijns, Frank J. Meye, and R. Jeroen Pasterkamp

Figure S1. FACS-based strategy to isolate GABAergic neurons from the mouse ventral midbrain and scRNA-seq control data. Related to Figure 1.

(A) Timeline of GABAergic neuron migration to the substantia nigra pars reticulata (SNr) and interpeduncular nucleus (IPN). Positioning of GABAergic neurons in the SNr requires interactions with mDAergic neurons and starts around E16.5 and is finalized around E18.5. **(B)** Fluorescence-activated cell sorting (FACS) gating strategy to select VGAT^{+} or VGAT^{+}Pitx3^{+} neurons from E16.5 or P0.5 *VGAT-Cre:Ai14:Pitx3-GFP* mice. FSC, forward-scattered light; SSC, side-scattered light.See STAR Methods for more details. **(C)** t-SNE embedding showing VGAT^{+} or VGAT^{+}Pitx3^{+} (boxed area) clusters as detected by the Louvain algorithm per single cell. **(D)** t-SNE embedding showing stage, plate-batch and plates data. In total, 14 brains from 6 litters of E16 embryos and 13 brains from 6 litters of P0 pups were used for scRNA-seq analysis. Nine plates were collected (see right panel) and VGAT^{+}Pitx3^{+} cells were collected to fill one entire plate for each developmental stage (E16.5 and P0.5). Therefore, while VGAT^{+}Pitx3^{+} cells normally represent approximately 2% of all VGAT^{+} cells they are overrepresented in the study due to more extensive sampling to aid their analysis. **(E, F)** Violin plots showing the distribution of cells across multiple features before (E) and after (F) filtering. A relatively high number of reads is detected per cell while the percentage of mitochondrial and ribosomal reads is relatively low, indicating that the quality of the cells is good. After filtering for cells with more than 2,000 genes 1,378 cells were removed. Additionally, 7,539 genes detected in fewer than 3 cells were filtered out. **(G)** Dot-plots showing the number of genes and the percentage of mitochondrial reads, ERCC reads, ribosomal reads and protein coding reads of the identified cells. After filtering, the percentage of mitochondrial and protein coding genes is comparable across ages. **(H)** Dot-plot showing unfiltered scaled and normalized expression of the top 4 differentially expressed genes (DEGs) per cluster calculated by Wilcoxon rank sum test (minimum normalized expression within cluster = 0.5, maximum normalized expression in other groups = 0.3, minimum fold change = 1,5). Disk size indicates positive fraction of cell within the group (%).

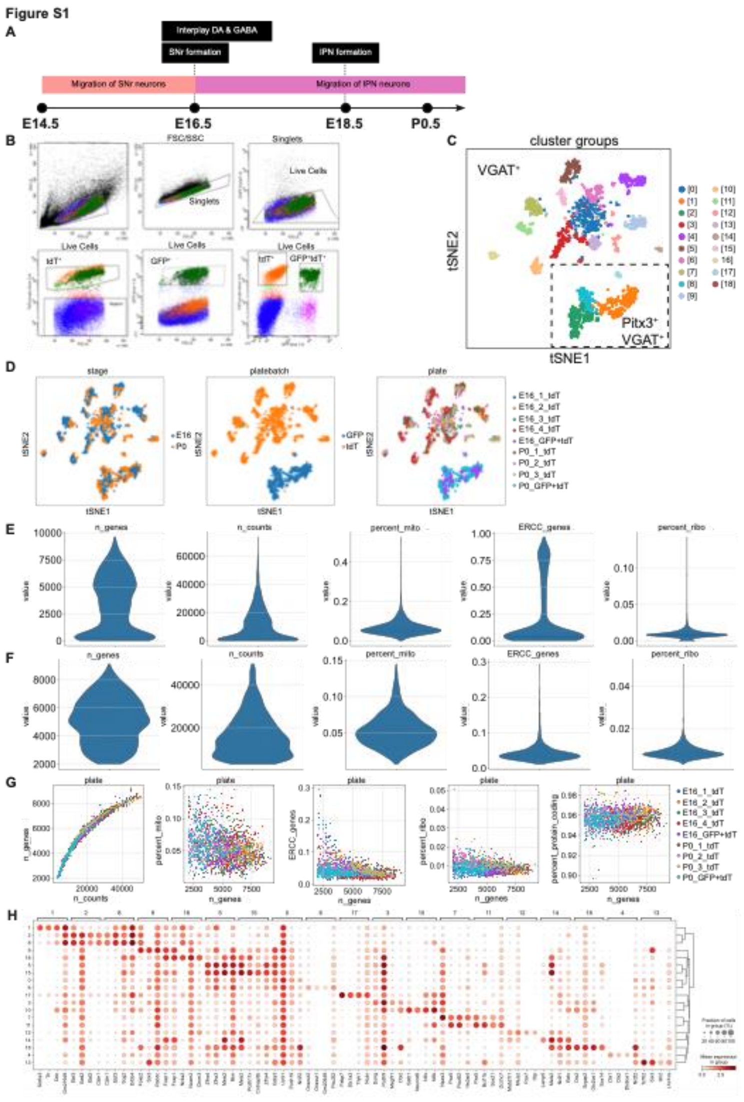

Figure S2. GABAergic neuron subtypes in the substantia nigra pars reticulata. Related to Figure 2. **(A)** Upper panels: *in situ* hybridization for *Pax5* and other substantia nigra pars reticulata (SNr) markers identified by (sub)clustering (Figure 2) on sagittal sections of the E18.5 ventral midbrain (from the Allen Brain Atlas). Dashed line indicates SNr. Arrows indicate labelling in caudal SNr. Rostral is to the left. c, caudal; d, dorsal; r, rostral; v, ventral; SNr, substantia nigra pars reticulata. Lower panels: UMAP embedding showing the expression of the selected (sub)cluster marker genes in the SNr subclusters. **(B)** *In situ* hybridization for different SNr markers identified by (sub)clustering (Figure 2) on sagittal sections of the postnatal ventral midbrain (from the Allen Brain Atlas). Dashed line indicates SNr. Rostral is to the left. dSNr, dorsal SNr; vSNr, ventral SNr. **(C)** *In situ* hybridization for different SNr markers identified by (sub)clustering (Figure 2) on coronal sections of the postnatal ventral midbrain (from the Allen Brain Atlas). Dashed line indicates SNr. VTA, ventral tegmental area. Scale bar, 100 μm.

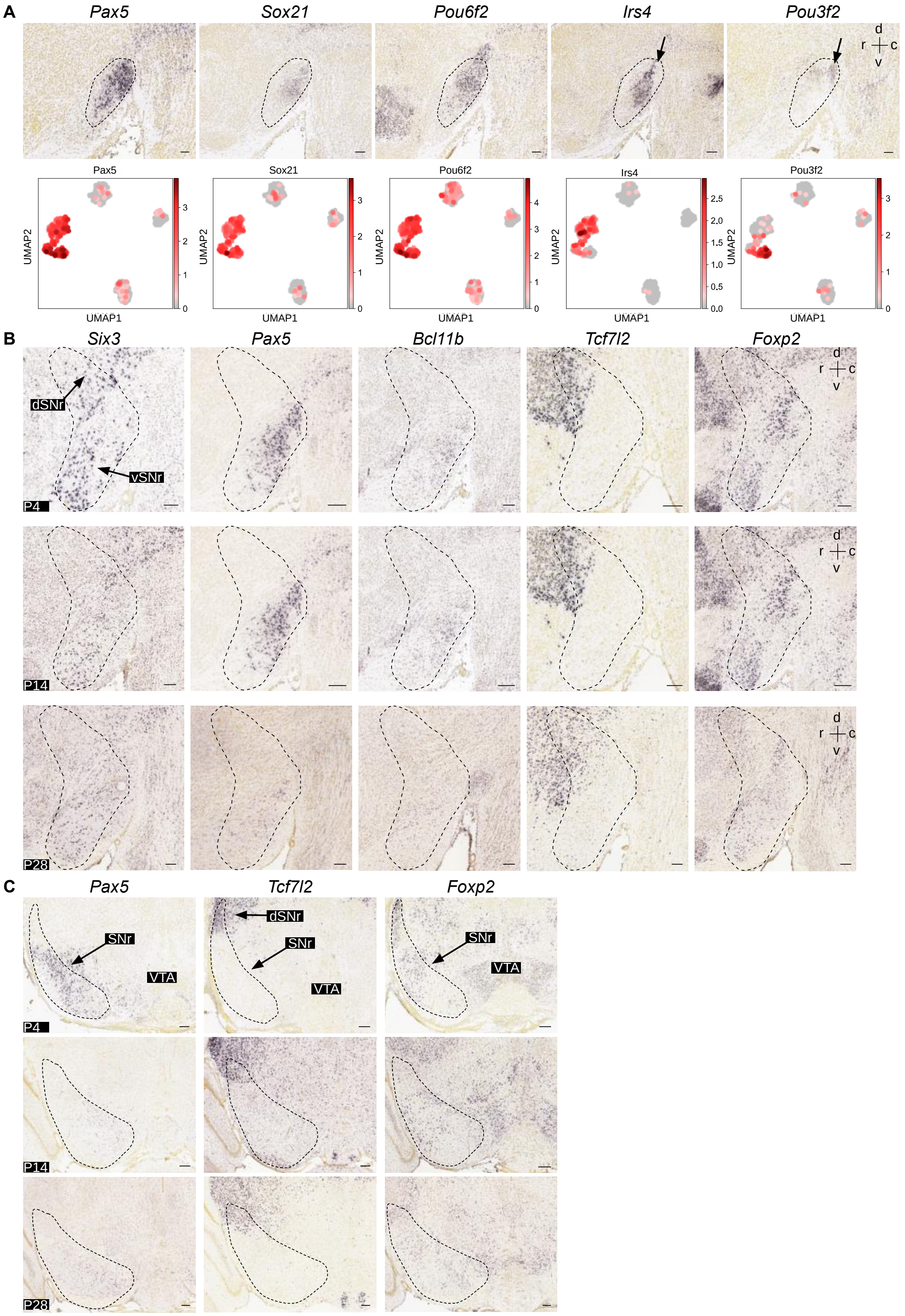

Figure S3. GABAergic neuron subtypes in the developing midbrain reticular formation and midbrain dopamine (mDA) system. Related to Figures 3 and 4.
(A) Double immunohistochemistry for tyrosine hydroxylase (TH) and Nkx2.2 on coronal sections of the ventral midbrain of E18.5 wildtype mice at rostral (left panel) and medial (right panel) levels. RN, red nucleus (indicated by dashed line); SNc, substantia nigra pars compacta. **(B)** Immunohistochemistry for TH, Nkx2.2 and tdTomato (tdT) on coronal sections of the ventral midbrain of E18.5 *VGAT-Cre:Ai14* mice. Dashed line indicates RN. Left panel: TH and Nkx2.2. labelling. Right panel: labelling for all three (TH, Nkx2.2, tdT) proteins. **(C-E)** *In situ* hybridization for *Nkx2.2* in sagittal (upper panels; rostral is to the left) and coronal (lower panels) sections of the ventral midbrain of P4, P14 or P28 mice (from the Allen Brain Atlas). Dashed lines indicate the SNr (upper panels) or mDA system (lower panels). c, caudal; d, dorsal; IPN, interpeduncular nucleus; MRF, midbrain reticular formation; r, rostral; v, ventral; VTA, ventral tegmental area. **(F-H)** *In situ* hybridization for *En1* in sagittal (upper panels; rostral is to the left) and coronal (lower panels) sections of the ventral midbrain of P4, P14 or P28 mice (from the Allen Brain Atlas). Dashed lines indicate the SNr (upper panels) or mDA system (lower panels). (**I**) Left panel: *in situ* hybridization for the hypothalamic marker *Dlx1* (subcluster 4) identified by (sub)clustering (Figure 4) on sagittal sections of the E18.5 ventral midbrain (from the Allen Brain Atlas). Dashed line indicates SNr. Rostral is to the left. Hy, Hypothalamus. Right panel: UMAP embedding showing the expression of *Dlx1* in the hypothalamic subcluster. (**J**) Left panel: *in situ* hybridization for hindbrain marker *Lamp5* (subcluster 14) identified by (sub)clustering (Figure 1) on sagittal sections of the E18.5 ventral midbrain (from the Allen Brain Atlas). Dashed line indicates SNr. Rostral is to the left. Hb, Hindbrain. Right panel: UMAP embedding showing the expression of *Lamp5* in the hindbrain subcluster. Scale bar, 100 µm.

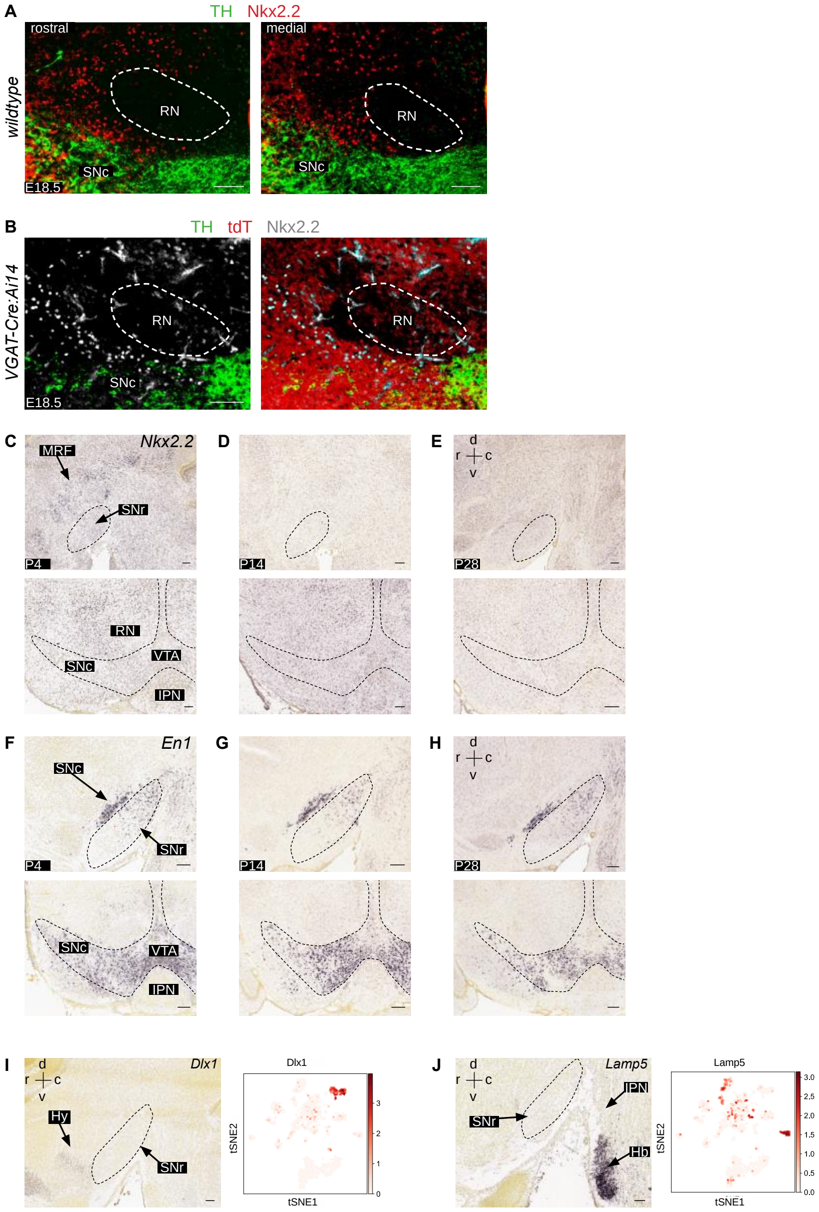

Figure S4. *In situ* hybridization for *Gad1* and *Gad2* and electrophysiological data. Related to Figure 5.
(A, B) *In situ* hybridization *Gad1* and *Gad2* in coronal sections of the ventral midbrain of P56 mice. VTA, ventral tegmental area; SNc, substantia nigra pars compacta; SNr, substantia nigra pars reticulata; IPN, interpeduncular nucleus; RN, red nucleus. Dashed line indicates mDA system and SNr. **(B-F)** Electrophysiological recordings of VGAT$^+$ and Pitx3$^+$ subtypes in the midbrain. **(B)** The action potential threshold is set more hyperpolarized for VGAT$^+$Pitx3$^+$ neurons compared to Pitx3$^+$ neurons (n VGAT$^+$ =13, n Pitx3$^+$ =12, n double VGAT$^+$Pitx3$^+$ =20, ANOVA, F(2.42)=4.2, p=0.034, Pitx3$^+$ vs VGAT$^+$Pitx3$^+$ p=0.03). Resting membrane potential (Vrest) **(C)**, Membrane resistance (RMembrane) **(D)**, Cellular capacitance **(E)** and voltage sag **(F)** are similar between VGAT$^+$, Pitx3$^+$ and VGAT$^+$Pitx3$^+$ neurons. Scale bar, 500 µm.

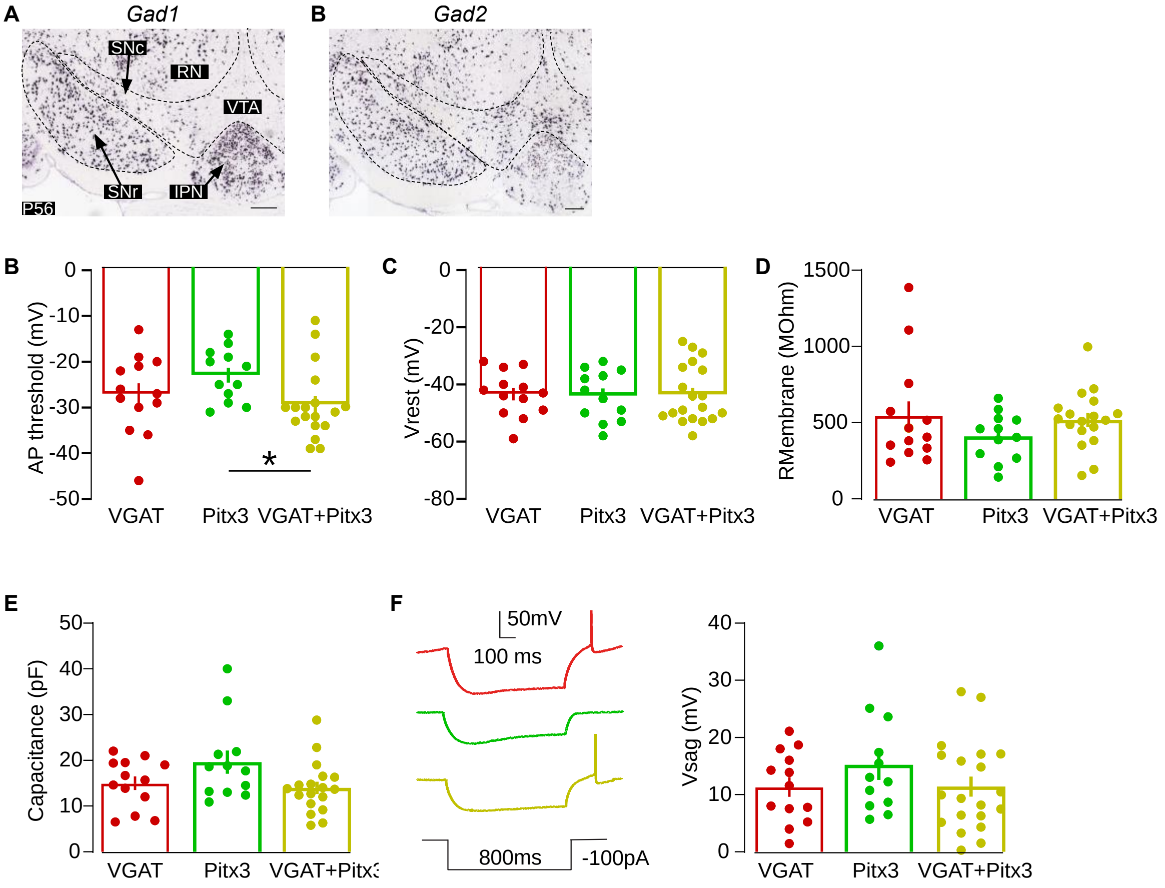

Figure S5. FLSM analysis of P5 *VGAT-Cre:Pitx3-FlpE:Ai65* mice. Related to Figure 5.

(A) Double immunohistochemistry for TH (green) and GFP (red) on coronal sections of E18.5 *Pitx3-FlpE:RCE* mice. Sections show ventral midbrain at medial levels. VTA, ventral tegmental area; RN, red nucleus; SNc, substantia nigra pars compacta. **(B)** Double immunohistochemistry for TH (green) and tdTomato (tdT, red) on coronal sections of the P0.5 *VGAT-Cre:Pitx3-FlpE:Ai65* mice. Sections show ventral midbrain at rostral (left panel), medial (middle panel), and caudal (right panel) levels. **(C, D)** Cell quantification in the DA neuron pool. Per mouse three sections obtained from three different rostral-caudal levels were used (n = 3 mice). Datapoints indicate sections and data are presented as means ±SEM. **(C)** Quantification of the number of TH^+ cells that express tdT. **(D)** Quantification of the number of tdT^+ cells that express TH^+. **(E-G)** P5 *VGAT-Cre:Pitx3-FlpE:Ai65* mice were immunostained for TH and tdT, optically cleared and imaged in a horizontal plane using fluorescent light sheet microscopy (FLSM). Representative max projection of 200 µm sections of a hemisphere from a horizontal plane z stack. Three different levels are shown to visualize all tdT^+ projections at P5 (schematics on the left). Boxed areas are shown at higher magnification on the right in colour and black/white. Boxed areas show higher magnification images of 50 µm sections in the cortex (CTX), lateral habenula (LHB), thalamus (Thal), striatum (STR), septum (Sept), and medial forebrain bundle (MFB): right panel: tdT, middle panel: TH, left panel: merged image. Dashed line indicates tdT labelling margins in the LHB, TH labelling in the medial habenula and labelling in the MFB. Stainings were performed on >3 mice with similar results. Scale bar, 100 µm.

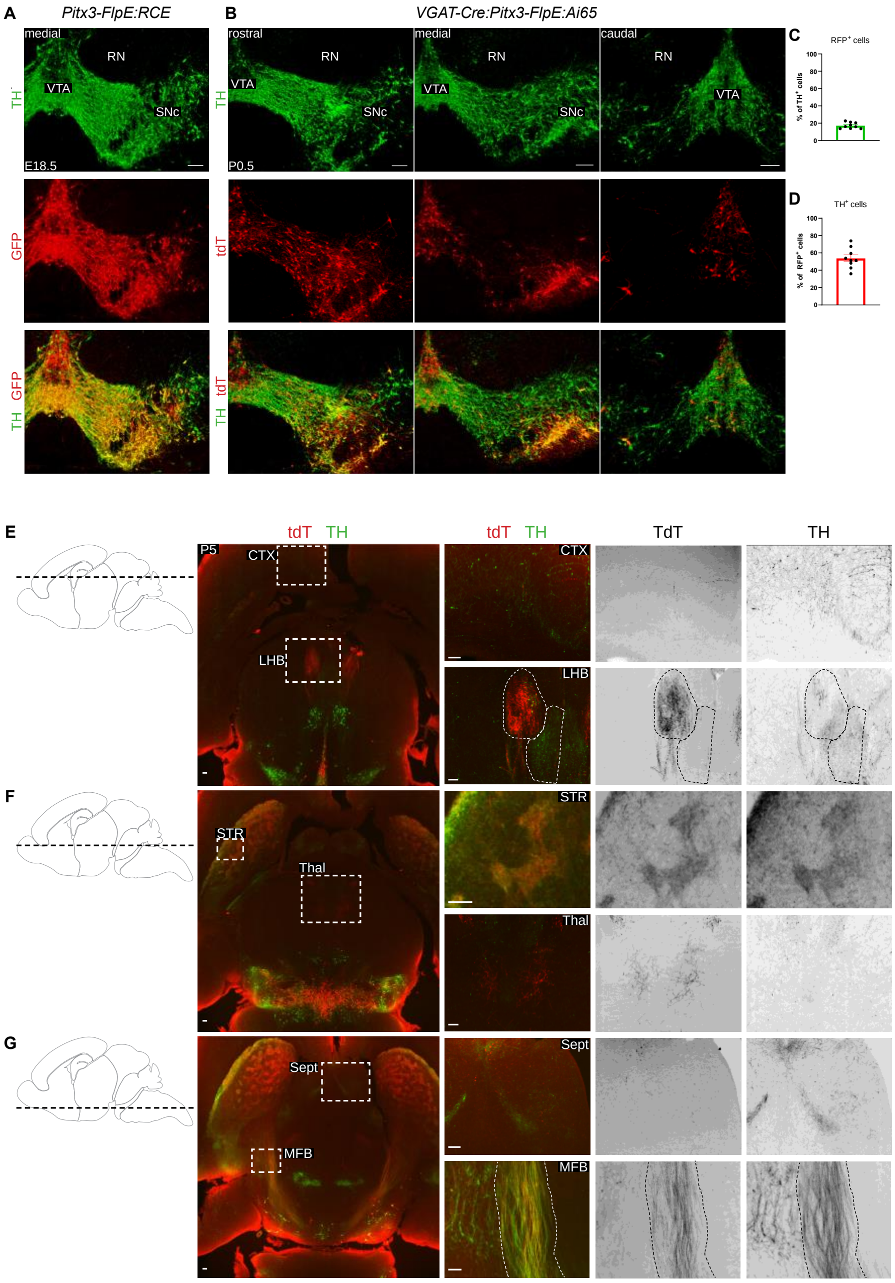

Figure S6. Ventral midbrain GABAergic neuron migration requires specific netrin-1 sources and receptors. Related to Figures 6 and 7.

(A-C) *in situ* hybridization for the GABAergic markers *Gad1*, *Gad2* and *Tal1* on coronal sections of the ventral midbrain of E18.5 *netrin-1*$^{+/-}$ and *netrin-1*$^{-/-}$ mice. Red dashed line indicates mDA system. Red arrows indicate ectopic cells in and around the SNc. RN, red nucleus; MRF, midbrain reticular formation; SNc, substantia nigra pars compacta; SNr, substantia nigra pars reticulata; VTA, ventral tegmental area. **(D-H)** Double immunohistochemistry for tyrosine hydroxylase (TH) and the indicated proteins on coronal sections of the ventral midbrain of E18.5 *netrin-1*$^{fl/fl}$ or *DTA* mice crossed with the indicated Cre lines. Each staining was performed on >3 embryos with similar results. Dashed line indicates border VTA and IPN. IPN, interpeduncular nucleus. **(I)** Dot-plot showing the expression levels of different netrin-1 receptors (deleted in colorectal cancer (DCC), down syndrome cell adhesion molecule (DSCAM), UNC5, a-integrins) in the different GABAergic clusters. Disk size indicates positive fraction of cell within the group (%). **(J)** Double immunohistochemistry for TH and Sox21 in coronal sections of the ventral midbrain of P0.5 *DCC*$^{-/-}$ embryos and littermate controls. SNr, substantia nigra pars reticulata. **(K)** Double immunohistochemistry for TH and Pax7 in coronal sections of the ventral midbrain of P0.5 *DSCAM*$^{-/-}$ embryos and littermate controls. Scale bar, 100 μm.

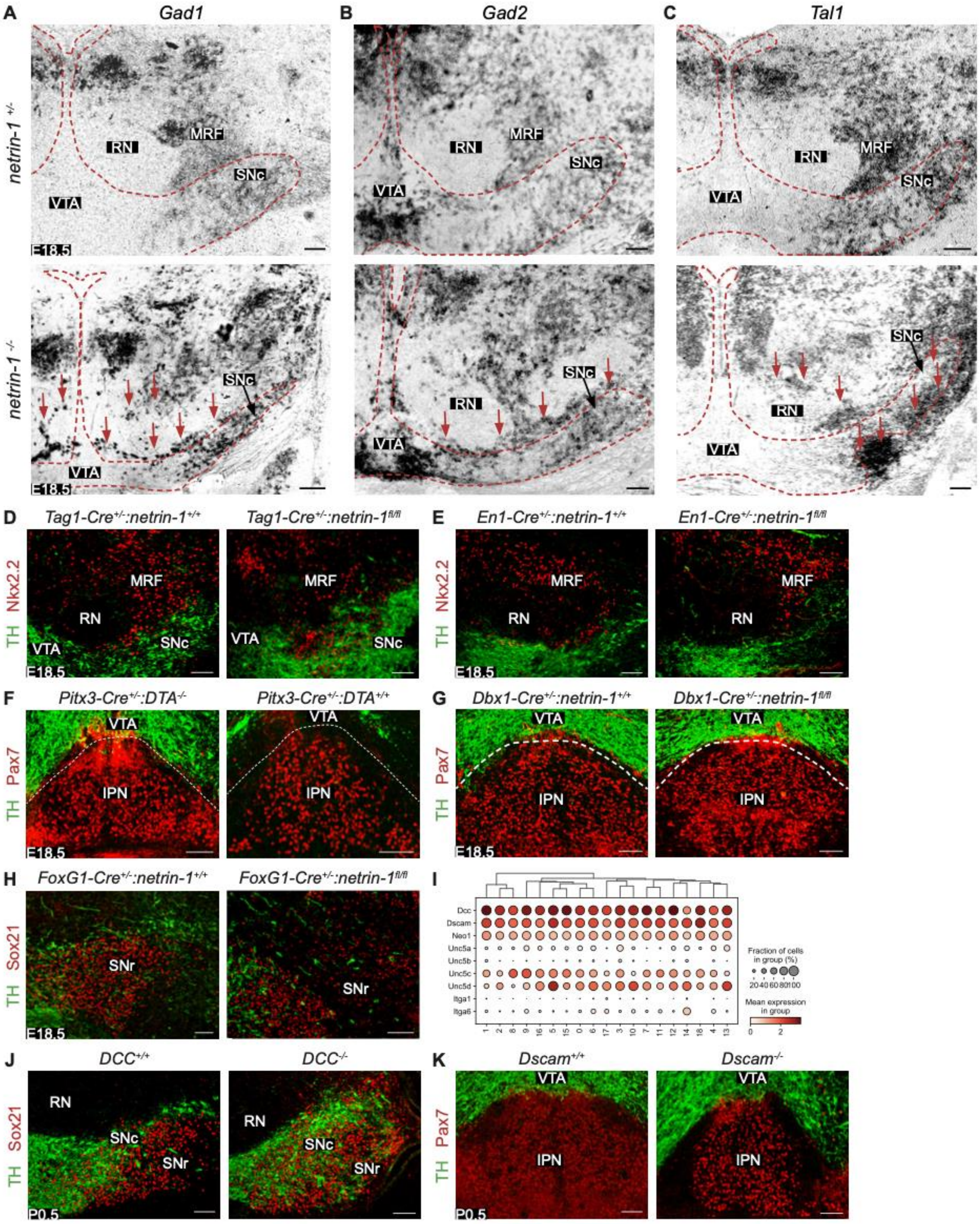

Table S1. Top 100 differentially expressed genes (DEGs) in scRNA-seq clusters, related to Figure 1 and S1.

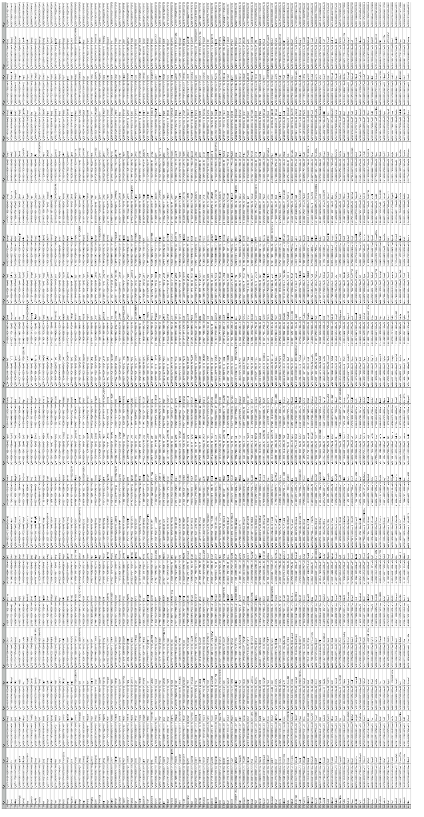

7

Table S2. Electrophysiological data VGAT, VGAT+Pitx3 and Pitx3 neurons. Related to Figure 5 and S4.

Figure	Variable	Group	N mice	N data points	Statistical test	Comparison	DF	Test value	P value	
5D	Action potentials (AP) per current step	VGAT	10	13	Repeated Measures Two-Way ANOVA	Cell type between subject	2,42	F=4.2	p=0.022	Tukey post h
		Pitx3		12						
		VGAT + Pitx3		20						
					Post-Hoc contrasts	DA vs GABA	2,42	mean i-j=-9.3	p=0.031	
						DA vs GABA-DA	2,42	mean i-j=-7.9	p=0.045	
S4B	AP threshold	VGAT	10	13	1 way ANOVA	Main effect cell type	2,42	F=3.7	p=0.034	
		Pitx3		12						
		VGAT + Pitx3		20						
					Post-Hoc contrasts	DA vs GABA	2,42	mean i-j=5.5	p=0.14	
						DA vs GABA-DA	2,42	mean i-j=6.98	p=0.03	
S4C	Vrest	VGAT	10	13	1 way ANOVA	Main effect cell type	2,42	F=0.11	p=0.98	
		Pitx3		12						
		VGAT + Pitx3		19						
S4D	Membrane resistance	VGAT	10	13	1 way ANOVA	Main effect cell type	2,40	F=1.1	p=0.3	
		Pitx3		12						
		VGAT + Pitx3		18						
S4E	Capacitance	VGAT	10	13	1 way ANOVA	Main effect cell type	2,40	F=2.8	p=0.07	
		Pitx3		12						
		VGAT + Pitx3		18						
S4F	Voltage sag	VGAT	10	13	1 way ANOVA	Main effect cell type	2,42	F=0.9	p=0.4	
		Pitx3		12						
		VGAT + Pitx3		20						
	Age of the animals at time of recording (PND)	VGAT	10	13	1 way ANOVA	main effect cell type	2,42	F=0.01	p=0.99	
		Pitx3		12						
		VGAT + Pitx3		20						

Table S3. Correlational analysis of age in postnatal days (over the P3-P12 range) with electrophysiological properties of the cells. Related to Figure 5 and S4.

		VGAT-Single Neurons		
**Measure**	**Pearson's R**	**P value unadjusted**	**Signif with alpha?**	**Signif with alpha adj?**
Age vs APs at 0 pA	0,1293	0,6737	No	No
Age vs APs at 100 pA	0,07568	0,8059	No	No
Age vs APs at 200 pA	0,1068	0,7285	No	No
Age vs Resting membrane potential	0,1961	0,5209	No	No
Age vs Membrane Resistance	-0,1758	0,5656	No	No
Age vs AP threshold	-0,5454	0,0539	No	No
Age vs Capacitance	-0,4028	0,1724	No	No
Age vs voltage sag -100 pA	-0,3025	0,3151	No	No
		**Pitx3-Single Neurons**		
**Measure**	**Pearson's R**	**P value unadjusted**	**Signif with alpha?**	**Signif with alpha adj?**
Age vs APs at 0 pA	0,2221	0,4878	No	No
Age vs APs at 100 pA	0,2929	0,3556	No	No
Age vs APs at 200 pA	0,5899	*0,0435*	*Yes*	No
Age vs Resting membrane potential	0,1094	0,735	No	No
Age vs Membrane Resistance	-0,2028	0,5272	No	No
Age vs AP threshold	-0,1802	0,5751	No	No
Age vs Capacitance	0,4962	0,1008	No	No
Age vs voltage sag -100 pA	-0,2836	0,3717	No	No
		**VGAT+ Pitx3 Neurons**		
**Measure**	**Pearson's R**	**P value unadjusted**	**Signif with alpha?**	**Signif with alpha adj?**
Age vs APs at 0 pA	0,04025	0,8662	No	No
Age vs APs at 100 pA	0,0502	0,8335	No	No
Age vs APs at 200 pA	0,2367	0,315	No	No
Age vs Resting membrane potential	0,3256	0,1873	No	No
Age vs Membrane Resistance	0,2089	0,4054	No	No
Age vs AP threshold	0,1037	0,6822	No	No
Age vs Capacitance	0,02051	0,9356	No	No
Age vs voltage sag -100 pA	-0,2655	0,2579	No	No

Table S4. Overview of netrin-1 receptors and cellular sources required for the migration of different GABAergic subtypes. Related to Figure 6, 7.

Subset	**SNr 1**	**3**	**IPN**	**VTA**	**MRF**
regional marker	Six3	Pax5, Sox21, Pou6f2, Bcl11b (Ctip2)	Pax7	Otx2	Nkx2.2
Netrin-1 receptor	Dscam	Dscam (data not shown)	DCC (data not shown)	DCC	DCC
Netrin-1 source	forebrain	forebrain (data not shown)	midbrain/rhombomere 1	midbrain/rhombomere 1	midbrain/rhombomere 1

Table S5. Overview of oligonucleotides. Related to the STAR Methods.

Genotyping			
**locus**	**forward primer**	**reverse primer**	**product size (bp)**
tdTomato WT	AAGGGAGCTGCAGTGGAGTA	CCGAAAATCTGTGGGAAGTC	279
tdTomato mutant	GGCATTAAAGCAGCGTATCC	CTGTTCCTGTACGGCATGG	196
VGAT-Cre WT	CTTCGTCATCGGCGGCATCTG	CAGGGCGATGTGGAATAGAAA	320
VGAT-Cre mutant	CTTCGTCATCGGCGGCATCTG	CCAAAAGACGGCAATATGGT	200
Foxg1-Cre WT	ACCCTGCCCTGTGAGTCTT	TTCTCCCACATTGCACCTC	310
Foxg1-Cre mutant	TAGTGAAACAGGGGCAATGG	TTCTCCCACATTGCACCTC	300
Netrin-1 KO WT	CTCAATAACCCGCACAACCT	CTCCGAGTCGTCTTCGTTCT	468
Netrin-1 KO mutant	CAGGTGGCAAGAGAAAAGGA	TCCGTTTGGATCTGGGATTA	432
Netrin-1 fl 2nd loxp site	TTGCAAGCCCTTCCACTACGA	AAGCAACTGTAAGGTCTCCGAAA	WT 241, MUT 375
DCC WT	TGGTCACCGTTGGTGTTCTC	GTGCGTGATGCAACCTTCTC	766
DCC mutant	CAAGACACATGGAAGGTGAAATG	CCCAAATCTTCTATATTACAATATC	374
Pitx3-Cre	TTCTCTGAACACACCTGGAAGAT	GATGTCCTTCACTCTGATTCTGG	450
HoxB1-Cre	TTCCCGCAGAACCTGAAGATGTTCG	GGGTGTTATAAGCAATCCCCAGAAATGC	320
DTA WT	CGTGATCTGCAACTCCAGTC	GGAGCGGGAGAAATGGATATG	462
DTA mutant	CGACCTGCAGGTCCTCG	CTCGAGTTTGTCCAATTATGTCAC	650
Pitx3-Flp WT	AAATTCAAACCACGCTCCTG	GTGTGTGTTTGTGCCTTTGG	366
Pitx3-Flp mutant	TTGTTGCTTTTTGCGTCTTG	GTGTGTGTTTGTGCCTTTGG	683
Tag1-Cre	TGAGTGCTTTAGCTCTACAGC	GACACAGCATTGGAGTCAGA	550
Dbx1-Cre WT	GAGGATGAGGAAATCACGGTG	GCAAGGAAATGTCTCTGGGAC	200
Dbx1-Cre mutant	GTCCAATTTACTGACCGTACACC	GTTATTCGGATCATCAGCTACACC	700
DSCAM WT	TCCTCCCTGTACGTTGTGTG	GATGGGCAAATGTCAAAGGT	398
DSCAM mutant	GCGAGATTAAGAACGAAC	TCCTCCTTGGTACGGGTA	400
RCE:FRT WT	CCCAAAGTCGCTCTGAGTTGTTATC	GAAGGAGCGGGAGAAATGGATATG	550
RCE:FRT mutant	CCCAAAGTCGCTCTGAGTTGTTATC	CCAGGCGGGCCATTTACCGTAAG	350
EIIa-cre	TTCCGCAGAACCTGAAGATGTTCG	GGGTGTTATAAGCAATCCCCAGAAAT	300
DCC fl	CAAGACACATGGAAGGTGAAATG	GACCTCACTTACATATCAAAATGG	WT 200, MUT 300

in situ hybridization probes		
**probe**	**Source**	**Method**
En1	cDNA on filter paper (kind gift from J. Partanen)	Vector-based
Tal1	cDNA on filter paper (kind gift from J. Partanen)	Vector-based
Gad1	cDNA on filter paper (kind gift from J. Partanen)	Vector-based
Pax5	Primers ABA	PCR based
Gad2	Primers ncbi primer blast	PCR based

in situ hybridization probes (PCR)				
**probe**	**primer_FW**	**primer_RV**	**product size (bp)**	**transcript**
Pax5	GTATTCAGGAGTCTCCAGTGCC	GAATACTGAGGGTGGCTGTAGG	451	NM_008782.1
Gad2	TCTTTTCTCCTGGTGGCG	TTGAGAGGCGGCTCATTC	870	NM_008078.2
